# BET Inhibition Silences Expression of *MYCN* and *BCL2* and Induces Cytotoxicity in Neuroblastoma Tumor Models

**DOI:** 10.1371/journal.pone.0072967

**Published:** 2013-08-23

**Authors:** Anastasia Wyce, Gopinath Ganji, Kimberly N. Smitheman, Chun-wa Chung, Susan Korenchuk, Yuchen Bai, Olena Barbash, BaoChau Le, Peter D. Craggs, Michael T. McCabe, Karen M. Kennedy-Wilson, Lydia V. Sanchez, Romain L. Gosmini, Nigel Parr, Charles F. McHugh, Dashyant Dhanak, Rab K. Prinjha, Kurt R. Auger, Peter J. Tummino

**Affiliations:** 1 Cancer Epigenetics Discovery Performance Unit, GlaxoSmithKline, Collegeville, Pennsylvania, United States of America; 2 Molecular Medicine Unit, GlaxoSmithKline, Collegeville, Pennsylvania, United States of America; 3 Platform Technology and Science, GlaxoSmithKline, Stevenage, United Kingdom; 4 Platform Technology and Science, GlaxoSmithKline, Collegeville, Pennsylvania, United States of America; 5 Lipid Metabolism Discovery Performance Unit, GlaxoSmithKline, Les Ulis, France; 6 Epinova Discovery Performance Unit, GlaxoSmithKline, Stevenage, United Kingdom; University of Navarra, Spain

## Abstract

BET family proteins are epigenetic regulators known to control expression of genes involved in cell growth and oncogenesis. Selective inhibitors of BET proteins exhibit potent anti-proliferative activity in a number of hematologic cancer models, in part through suppression of the *MYC* oncogene and downstream Myc-driven pathways. However, little is currently known about the activity of BET inhibitors in solid tumor models, and whether down-regulation of MYC family genes contributes to sensitivity. Here we provide evidence for potent BET inhibitor activity in neuroblastoma, a pediatric solid tumor associated with a high frequency of *MYCN* amplifications. We treated a panel of neuroblastoma cell lines with a novel small molecule inhibitor of BET proteins, GSK1324726A (I-BET726), and observed potent growth inhibition and cytotoxicity in most cell lines irrespective of *MYCN* copy number or expression level. Gene expression analyses in neuroblastoma cell lines suggest a role of BET inhibition in apoptosis, signaling, and N-Myc-driven pathways, including the direct suppression of *BCL2* and *MYCN*. Reversal of *MYCN* or *BCL2* suppression reduces the potency of I-BET726-induced cytotoxicity in a cell line-specific manner; however, neither factor fully accounts for I-BET726 sensitivity. Oral administration of I-BET726 to mouse xenograft models of human neuroblastoma results in tumor growth inhibition and down-regulation *MYCN* and *BCL2* expression, suggesting a potential role for these genes in tumor growth. Taken together, our data highlight the potential of BET inhibitors as novel therapeutics for neuroblastoma, and suggest that sensitivity is driven by pleiotropic effects on cell growth and apoptotic pathways in a context-specific manner.

## Introduction

Aberrant epigenetic regulation of transcription is a common hallmark in cancer and other diseases [1]. Therapeutic agents targeting chromatin “writers” (e.g. histone methyltransferases) and “erasers” (e.g. histone deacetylases) have been developed [1]; however, the therapeutic potential of chromatin “readers” has remained largely unexplored. Chromatin readers bind to specific modifications on histone tails, translating the histone “code” into transcriptional effects by recruiting co-activator or co-repressor complexes to target genes [2].

The bromodomain and extra-terminal (BET) family of proteins, including BRD2, BRD3, BRD4, and BRDT, are chromatin reader proteins that bind via tandem bromodomains to acetylated lysines in histone N-terminal tails [3]. BET proteins recruit co-activator complexes to chromatin to promote transcription of target genes. BRD4 regulates a number of genes essential for cell growth through the recruitment and maintenance of the pTEFb complex at gene promoters during mitosis [4,5]. BRD2 interacts with a number of transcription factors, including E2F family members, and regulates the expression of several E2F-dependent cell cycle genes [6,7]. While less is known about BRD3 and the testis-specific BRDT, both proteins bind to acetylated histones to promote transcription of growth-associated genes (BRD3) or chromatin remodeling (BRDT) [8,9].

Selective inhibitors that specifically disrupt the interaction between BET proteins and acetylated histones were recently described [10–14]. Initial evidence for the therapeutic potential of BET inhibitors in cancer was observed in models of NUT midline carcinoma (NMC) [12], a rare but lethal malignancy characterized by chromosomal translocations that express a fusion protein encoded by the bromodomains of BRD4 (or less frequently, BRD3) and the *NUT* locus [15]. BET inhibition resulted in proliferation arrest and spontaneous differentiation in NMC cell lines, as well as tumor growth inhibition in murine NMC xenograft models [12]. Additionally, potent anti-proliferative activity has been observed with a number of BET inhibitors in models of hematologic cancer, including acute myeloid leukemia [16,17], MLL-fusion leukemias [11], Burkitt’s lymphoma [17], multiple myeloma [18], and B-cell acute lymphoblastic leukemia [19]. Regulation of Myc driven transcription programs was cited as a consequence of BET inhibition in these tumor models, with BET inhibitors directly silencing *MYC* gene expression via disruption of BET protein binding at the *MYC* locus [11,16–18].

MYC-family transcription factors, including Myc, N-Myc, and L-Myc, are key regulators of cell growth and survival [20]. *MYC* gene amplification is one of the most common copy-number alterations observed in cancer [21], and over-expression or translocation of the *MYC* locus is known to contribute to deregulated Myc activity. Myc plays an important role in hematologic cancers as well as a number of solid tumors including breast, lung, bladder, and colon cancer [22]. Amplification or over-expression of *MYCN* or *MYCL1* is frequently observed in lung cancer (*MYCN*, *MYCL1*), ovarian cancer (*MYCL1*), breast cancer (*MYCN*), and cancers of neural origin including glioblastoma (*MYCL1*, *MYCN*), medulloblastoma (*MYCN*), and neuroblastoma (*MYCN*) [22]. Despite the well-established role of MYC family proteins in driving cancer cell growth, no direct MYC-targeted therapeutic agent has advanced to clinical studies [20,23].

Given the potential therapeutic benefit of MYC-family transcription factor inhibition in a wide variety of cancers, we investigated the effects of BET inhibition in neuroblastoma, an aggressive pediatric cancer associated with a high frequency of *MYCN* gene amplification. Herein, we report the results of our studies using GSK1324726A (I-BET726), a novel, potent, and selective small molecule inhibitor of BET proteins.

## Results

### I-BET726 is a selective small molecule inhibitor of BET proteins

**Figure 1:**
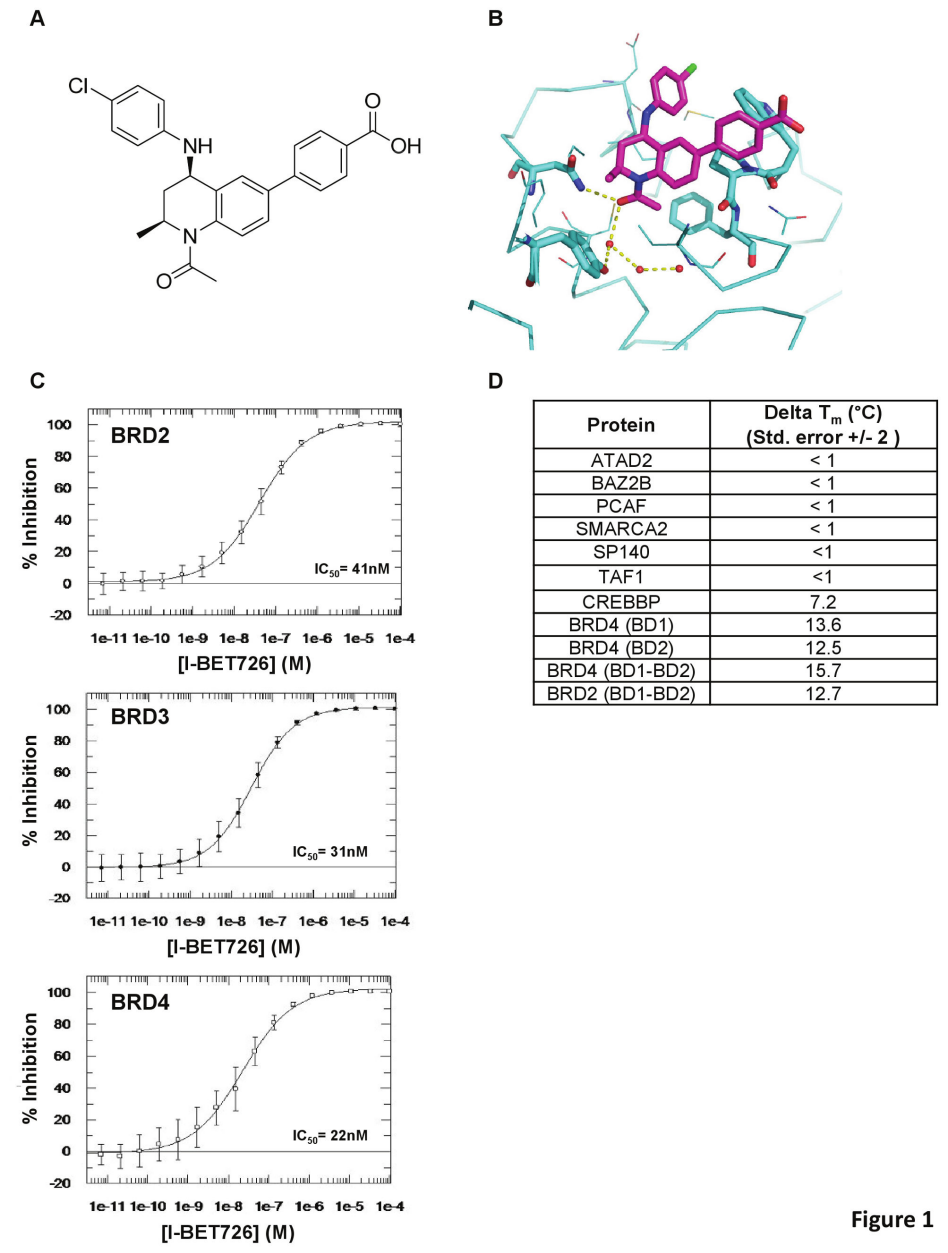
I-BET726: a novel selective inhibitor of BET family proteins. (**a**) Chemical structure of GSK1324726A (I-BET726). (**b**) Crystal structure of I-BET726 (magenta) bound to the acetyl-binding pocket of BRD4-BD1 (resolution: 1.6 Å). (**c**) Concentration response curves for determination of binding affinity of I-BET726 to BRD2, BRD3, and BRD4 bromodomains by ligand displacement detected using Time Resolved Fluorescence Resonance Energy Transfer (TR-FRET). IC_50_ values for BRD2, BRD3, and BRD4 are indicated. (**d**) Selectivity profile of I-BET726 showing average temperature shifts (delta T_m_) in degrees Celsius for a panel of bromodomain proteins using a fluorescent thermal shift assay. N= 2 for all proteins except CREBBP (n= 4).

I-BET726 is a novel small molecule inhibitor (Figure 1A) that binds to the acetyl-lysine recognition pocket of BET family proteins (Figure 1B). It binds with high affinity to BRD2 (IC_50_= 41 nM), BRD3 (IC_50_= 31 nM), and BRD4 (IC_50_= 22 nM), and competes with tetra-acetylated histone H4 peptides (K5ac, K8ac, K12ac, K16ac) for binding to the bromodomains of these proteins (Figure 1B, 1C). I-BET726 is highly selective for BET family proteins (Figure 1D), exhibiting no binding affinity for any bromodomain-containing homolog tested with the exception of CREBBP, for which I-BET726 binds with >1000-fold lower affinity than to BET family proteins (Figure S1 in File S1).

### I-BET726 Inhibits Cell Growth and Induces Cytotoxicity in Neuroblastoma Cell Lines

**Figure 2:**
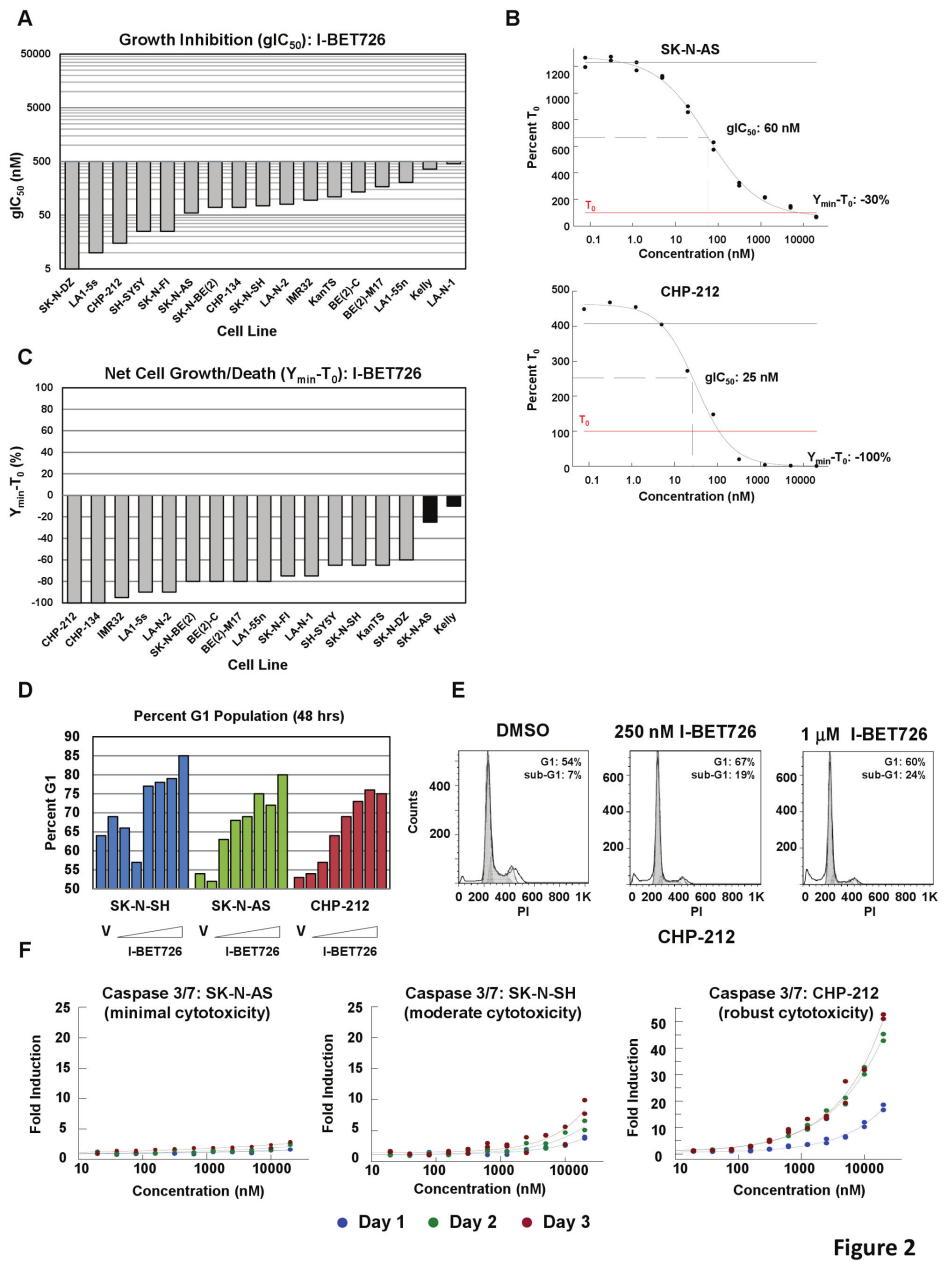
I-BET726 treatment results in potent growth inhibition and cytotoxicity in neuroblastoma cell lines. (**a**) gIC_50_ values observed for I-BET726 in a panel of neuroblastoma cell lines obtained from a 6 day growth-death assay. (**b**) Concentration response curves for SK–N-AS and CHP-212 from 6 day growth-death assay. Black horizontal line indicates growth in DMSO-treated controls. Red line indicates T_0_ value (100%). gIC_50_ and Y_min_-T_0_ values are indicated. Data presented as the average of two independent curves from a single experiment, and is representative of data from three independent biological replicates. (**c**) Y_min_-T_0_ values observed for I-BET726 in the panel of neuroblastoma cell lines obtained from a 6 day growth-death assay. Gray bars indicate a cytotoxic response, defined by a Y_min_-T_0_ value ≤ -50, with evidence of net cell death (Y < T_0_) along the growth curve at concentrations less than 6 µM. (**d**) Graph summarizing number of cells in G1 phase (as a percent of total cell population) in the indicated neuroblastoma cell lines following treatment with a titration of I-BET726 (5 nM-20 000 nM) for 2 days based on propidium iodide staining. V represents vehicle (DMSO) control sample. (**e**) Histograms generated from cell cycle analysis in the CHP-212 cell line following 4 days treatment with the indicated concentration of I-BET726. Percentage of cells in G1 phase and sub-G1 phase are indicated. (**f**) Caspase induction in the indicated neuroblastoma cell lines following treatment with a titration of I-BET726 for one, two, or three days. Data is presented as fold induction over DMSO controls, following normalization to total cell number as measured by CellTiter-Glo.

Since potent anti-proliferative activity was observed for BET inhibitors in *MYC*-driven hematologic cancer models, we screened a panel of neuroblastoma cell lines, in which *MYCN* amplification is common, for effects on cell growth following I-BET726 treatment. All neuroblastoma cell lines tested exhibited potent growth inhibition, with a median growth IC_50_ value (gIC_50_; inhibitor concentration resulting in 50% growth inhibition) equal to 75 nM (Figure 2A; Table S1). Analysis of I-BET726 in other solid tumor cell lines revealed some level of anti-proliferative activity in most, but not all, cell lines tested (Figure S2A in File S1), which is consistent with previous reports on another BET inhibitor [17]. However, growth inhibition in neuroblastoma cell lines was more potent and consistent than effects observed in any other solid tumor model, suggesting that neuroblastoma cell lines are particularly sensitive to BET inhibition. A similar pattern of growth inhibition in neuroblastoma was observed with another BET inhibitor, I-BET151 (Figure S2B in File S1) [11], albeit at a potency about 5-fold lower than I-BET726 (Figure S2C in File S1). This shift in cellular potency between I-BET726 and I-BET151 is consistent with observations in other solid and hematologic cancer cell lines models (data not shown). Potent growth inhibition with I-BET726 was observed irrespective of *MYCN* amplification status (Table S1) or level of *MYC* or *MYCN* expression (Figure S3 in File S1). Additionally, we observed no correlation between sensitivity and expression of BRD2, BRD3, or BRD4 (Figure S4 in File S1).

Closer examination of the growth curves for neuroblastoma cell lines, plotted as a percent of the T_0_ value, revealed that I-BET726 triggers net cell death, with concentration response curves falling below the T_0_ measurement (Figure 2B). To examine the cell death response in more detail we determined Y_min_-T_0_ values for each growth curve. Y_min_-T_0_ values are calculated by subtracting the T_0_ measurement (set at 100%) from the Y-value at the bottom of the growth curve, thus providing a measure of net cell growth (positive Y_min_-T_0_) or net cell death (negative Y_min_-T_0_) at the assay end point. Analysis of average Y_min_-T_0_ values in the neuroblastoma cell line panel indicated that all cell lines tested exhibited some level of net cell death in response to I-BET726 (Figure 2C). Fifteen out of seventeen cell lines exhibited >50% net cell death (Y_min_-T_0_≤ -50%), with evidence of net cell death (Y < T_0_ value) occurring at concentrations of compound below 6 µM (Figure 2C). Similar responses were obtained using other measures of proliferation (Figure S5 in File S1). The cell death response observed in neuroblastoma was more potent and consistent across cell lines compared to other solid tumor models, again suggesting that neuroblastoma cell lines are more broadly sensitive to BET inhibition (Figure S6A in File S1). Importantly, a similar pattern of net cell death was observed in the neuroblastoma cell line panel following treatment with the BET inhibitor I-BET151 (Figure S6B in File S1), suggesting that the cell death response is an on-target effect of BET inhibition. No significant correlation was observed between *MYC* or *MYCN* expression and cell death response to either I-BET compound (Figure S7 in File S1).

To investigate the mechanism of action of I-BET726 in neuroblastoma, we examined changes in cell cycle progression in three cell lines exhibiting variable responses to I-BET726: SK–N-AS (minimal net cell death), SK–N–SH (moderate net cell death), and CHP-212 (robust net cell death). I-BET726 treatment resulted in a concentration-dependent induction of G_1_ arrest in all three cell lines by 48 hours (Figure 2D). Analysis following four days of treatment revealed an increase in the sub-G_1_ fraction in CHP-212 cells in the presence of 250 nM or 1 µM I-BET726 (Figure 2E), but not in SK–N-AS or SK–N–SH (data not shown). This observation is consistent with the markedly higher concentration of I-BET726 required in these cell lines to observe net cell death compared to CHP-212 (Figure 2B, Figure S8 in File S1) in the 6 day growth-death assay.

Caspase 3/7 induction was observed in SK–N–SH and CHP-212 cells as early as 24 hours post-treatment (Figure 2F). Caspase induction was concentration- and time-dependent, and qualitatively tracked with the relative amount of cytotoxicity observed in the two cell lines in the 6 day growth-death assay (Figure 2B, Figure S8 in File S1). In contrast, minimal caspase activation was observed in the SK–N-AS cell line at any time point assayed (Figure 2F). Analysis of additional cell lines revealed induction of caspase activity after 3 days of treatment in all cases where potent net cell death was observed in the 6 day growth-death assay (Figure S9 in File S1). Thus, apoptosis is specifically triggered in the subset of cell lines that exhibit a cytotoxic response to I-BET726.

### I-BET726 modulates expression of genes involved in apoptosis, signaling, and MYC-family pathways

**Figure 3:**
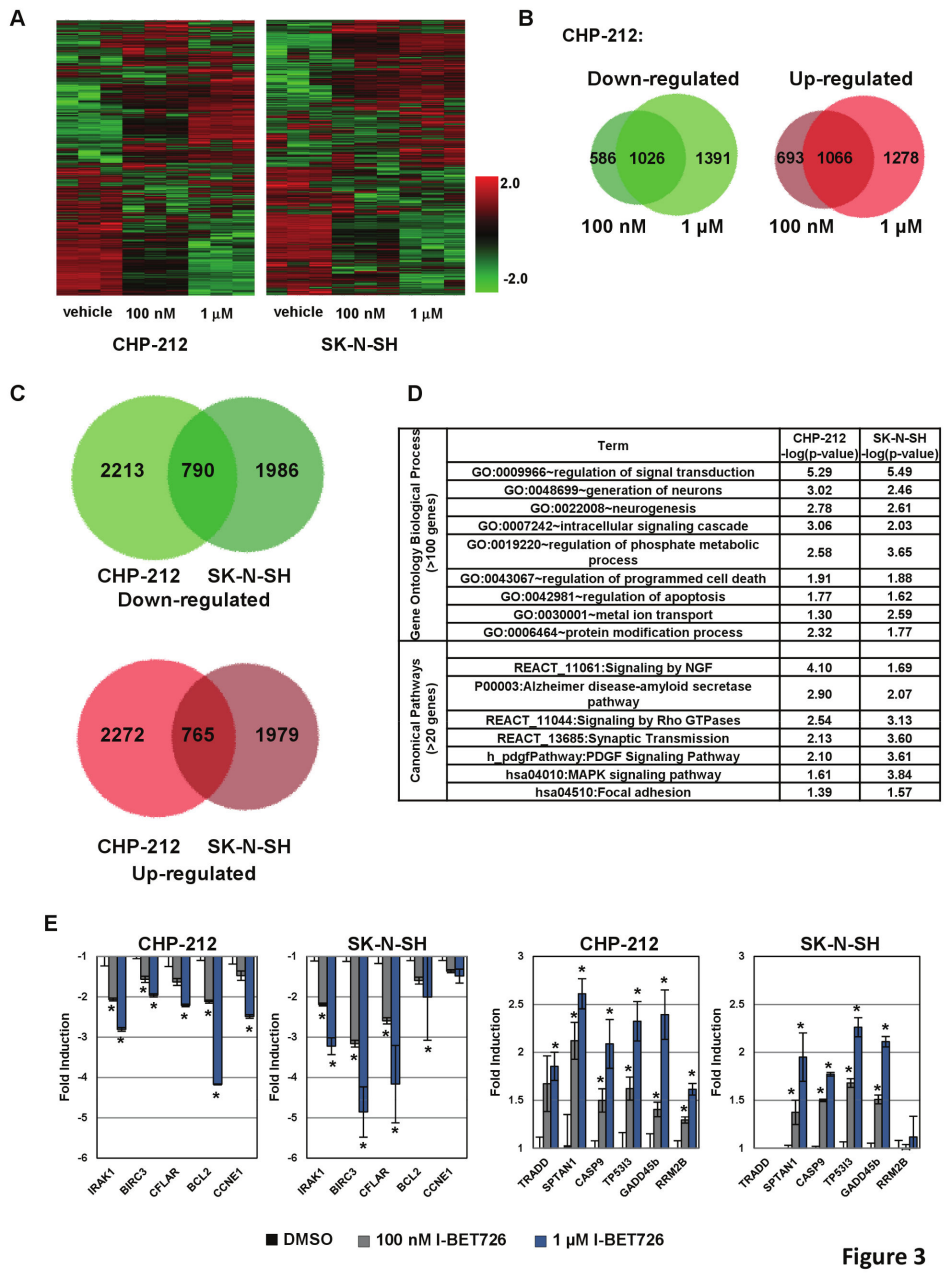
Global transcript profiling in neuroblastoma cell lines treated with I-BET726 reveals gene expression changes in apoptotic and signaling pathways. (**a**) Hierarchical clustering of statistically significant probes that were differentially expressed in 100 nM or 1 µM treatments of I-BET726 relative to vehicle in CHP-212 and SK–N–SH. (**b**) Venn analysis for up-regulated and down-regulated probes described in (a) in the CHP-212 cell line. (**c**) Venn analysis for overlap of up-regulated and down-regulated probes in the SK–N–SH and CHP-212 cell lines. (**d**) Functional analyses of expression changes were performed by GO and canonical pathway enrichment at the gene level. A subset of statistically significant categories for GO Biological Process (>100 genes) and canonical pathways (>20 genes) from KEGG and BioCarta that were common among the two cell lines are shown. (**e**) qRT-PCR confirmation of a subset of genes selected from the functional analyses described in (d). Data represent mean value ± standard deviation for three independent biological replicates. Asterisks indicate statistical significance as measured by t-test (p <0.05).

To gain insight into the transcriptional changes induced by I-BET726 in neuroblastoma, *MYCN-*amplified (CHP-212) and non-*MYCN-*amplified (SK–N–SH) cell lines were treated with 100 nM or 1 µM I-BET726 for 16 hours and profiled by Illumina microarrays. A total of 6040 (CHP-212) or 5520 (SK–N–SH) probes exhibited significant differential expression upon treatment with either concentration of compound (Figure 3A, Table S2). In CHP-212, 3003 of these probes decreased in expression and 3037 increased in expression, with a large degree of overlap between the two treatment groups (Figure 3B). Similar patterns of concentration-dependent up- and down-regulation were observed in the SK–N–SH cell line (Figure S10 in File S1). There were 765 and 790 common up- and down-regulated genes, respectively, between the two cell lines (Figure 3C).

To understand the biological relevance of these expression changes, we performed functional analyses by Gene Ontology Biological Process (GO BP) and canonical pathway enrichment (Figure 3D, Table S3). Consistent with the potent cytotoxicity observed in these cell lines upon prolonged treatment with I-BET726, we observed a statistical over-representation of cell death, apoptosis, and signal transduction pathways by GO BP analyses in both cell lines. Furthermore, BioCarta and KEGG (Kyoto Encyclopedia of Genes and Genomes) pathway analyses identified a number of signaling pathways, including MAPK, PDGF, and NGF, that are significantly enriched in the two data sets (Figure 3D, Table S3). Concentration-dependent expression changes in a subset of genes involved in apoptosis and signaling were confirmed by qRT-PCR following I-BET726 (Figure 3E) and I-BET151 (Figure S11A in File S1) treatment, further suggesting a role for these pathways in mediating cellular response to BET inhibition.

**Figure 4:**
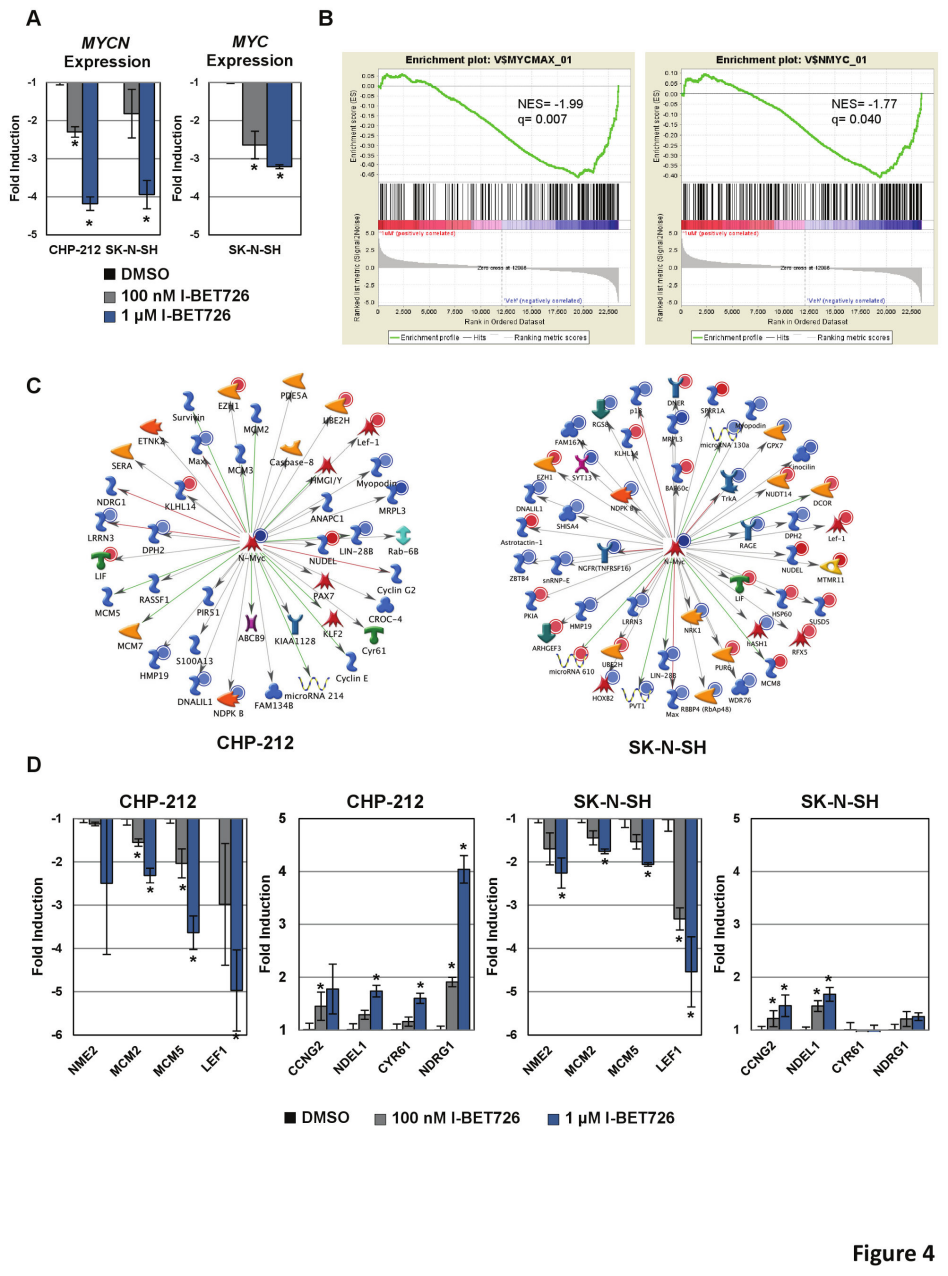
Global transcript profiling reveals gene expression changes in MYC-family pathways. (**a**) qRT-PCR confirmation of changes in *MYCN* and *MYC* expression following treatment with the indicated concentration of I-BET726. Data represent mean value ± standard deviation for three independent biological replicates. Asterisks indicate statistical significance as measured by t-test (p <0.05). (**b**) GSEA enrichment plots showing the down-regulation of gene sets associated with Myc/Max and N-Myc binding motifs in I-BET726-treated CHP-212 cells. Normalized enrichment scores (NES) and FDR q values are indicated. (**c**) A *MYCN* transcriptional regulation network was constructed (see Materials and Methods) to depict N-Myc pathway genes that were modulated by I-BET726 treatment. Red and blue circles represent increased and decreased expression changes, respectively. Green, red and grey edges are shown for activation, inhibition and unspecified interaction types, respectively. (**d**) qRT-PCR confirmation of a subset of genes selected from the N-Myc network analysis described in (c). Data presented as described in (a).

We observed decreased expression of MYC-family genes in both cell lines in the microarray dataset. Concentration-dependent silencing of *MYCN* following I-BET726 treatment was confirmed by qRT-PCR in both cell lines. *MYC* suppression was only observed in SK–N–SH, as CHP-212 cells do not express detectable levels of *MYC* (Figure 4A; Figure S3 in File S1). Similar changes in expression of *MYCN* and *MYC* were observed following I-BET151 treatment (Figure S11B in File S1), further confirming a role for BET proteins in regulating expression of these genes. Consistent with MYC-family gene suppression, Gene Set Enrichment Analyses (GSEA) revealed significant down-regulation of genes associated with *MYC* and *MYCN* binding motifs, as well as a number of MYC-family transcriptional signatures in both cell lines following I-BET726 treatment (Figure 4B; Table S4). The transcriptional profiles of *MYC* and *MYCN* are thought to be largely redundant [24–28]; thus, suppression of both *MYC* and *MYCN* likely contribute to the down-regulation of *MYC/MYCN* targets in SK–N–SH, whereas in CHP-212 *MYCN* suppression likely accounts for these effects. A larger number of *MYC*-associated signatures were significantly down-regulated in CHP-212 compared to SK–N–SH (Table S4), perhaps reflecting the reduced expression of MYC-family genes and induction of Myc/N-Myc driven pathways in the non-amplified cell line. Nonetheless, it appears that I-BET726 suppresses pathways associated with MYC family genes in neuroblastoma independent of amplification status.

To further investigate the effects of I-BET726 on N-Myc pathways, an N-Myc transcriptional regulation network was constructed and filtered to visualize transcriptional changes seen in our Dataset 42 genes from the N-Myc transcriptional regulation network were regulated by I-BET726 in CHP-212 and 50 were observed in the gene lists for SK–N–SH, with an overlap of 16 genes between the two cell lines (Figure 4C; Table S5). Concentration-dependent changes in expression were confirmed by qRT-PCR for a number of these N-Myc network genes in the two cell lines with I-BET726 (Figure 4D) and I-BET151 (Figure S11C in in File S1). Of the 8 genes validated by qRT-PCR, 5 are also known to be regulated by Myc [29]; thus, in SK–N–SH down-regulation of both *MYC* and *MYCN* may contribute to these gene expression changes.

### I-BET726 directly inhibits *MYCN* expression

**Figure 5:**
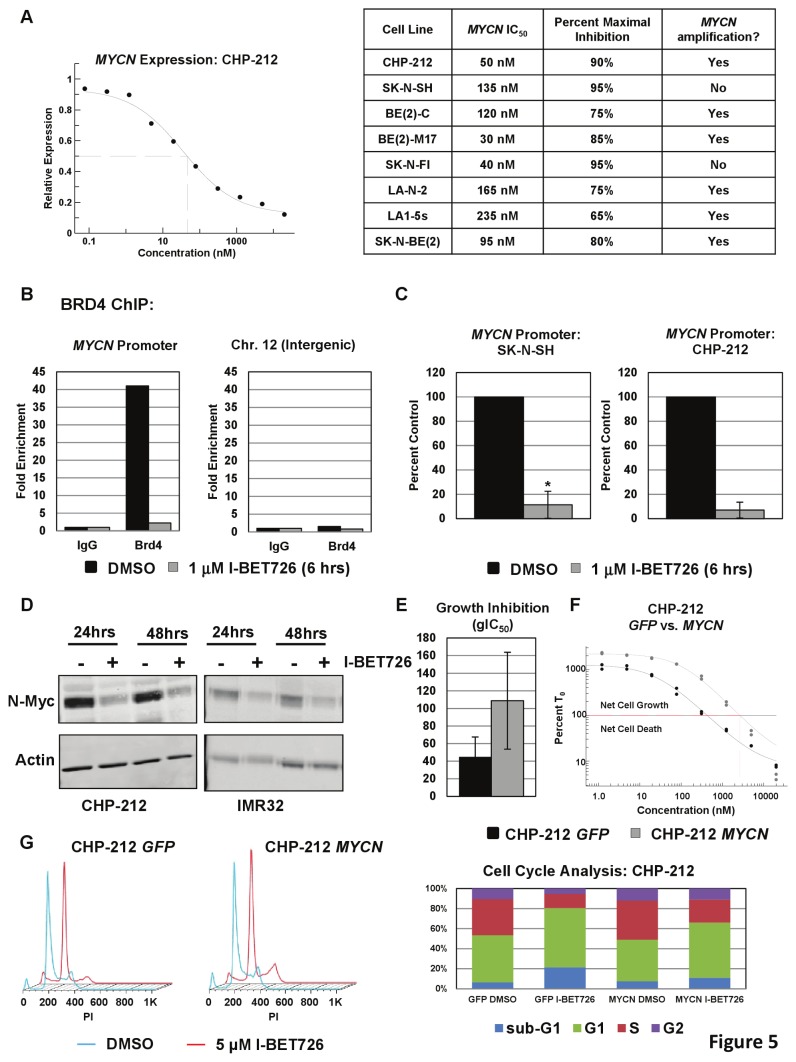
*MYCN* expression is directly regulated by BRD4 and repressed by treatment with I-BET726. (**a**) Left: Concentration response curve for *MYCN* RNA expression following 24 hour treatment with I-BET726 in the CHP-212 cell line. Data was normalized to GAPDH and is presented as expression relative to DMSO-treated controls. Data presented as the average of two independent curves from a single experiment, and is representative of data from three independent biological replicates. Right: Table of IC_50_ and percent inhibition values for *MYCN* suppression following 24 hour treatment with I-BET726 in the indicated cell lines. (**b**) BRD4 ChIP in the non-*MYCN*-amplified cell line SK–N–SH. Binding of BRD4 to the *MYCN* promoter or to an intergenic region on Chromosome 12 following treatment with vehicle or 1µM I-BET726 for six hours. Data is presented as fold enrichment over signal generated from IgG control immunoprecipitations. Data shown is from a single experiment representative of typical results. (**c**) BRD4 ChIP data at the *MYCN* promoter, presented as percent of vehicle control signal in the non-*MYCN*-amplified cell line SK–N–SH (left) and the *MYCN*-amplified cell line CHP-212 (right). SK–N–SH data represents the mean value ± standard deviation for three independent biological replicates. Asterisk indicates statistical significance as measured by t-test (p= 0.005). CHP-212 data represents the mean value ± standard deviation for two independent biological replicates. (**d**) Western blot analysis of N-Myc expression in the *MYCN*-amplified cell lines CHP-212 and IMR32 following 24 or 48 hour treatment with vehicle or 1µM I-BET726. Actin expression included as a loading control. (**e**) gIC_50_ values obtained from CHP-212 cells overexpressing *GFP* or *MYCN* following treatment with I-BET726 in a 6 day growth-death assay. Data represents the mean value ± standard deviation from four independent experiments. (**f**) Concentration response curves for *GFP* or *MYCN*–overexpressing CHP-212 cells from a 6 day growth-death assay. Horizontal line indicates T_0_ measurement (normalized to 100%). Data shown was from a single experiment representative of typical results. (**g**) Left: Histograms generated from cell cycle analysis in *GFP*- or *MYCN*-overexpressing CHP-212 cells following 4 days treatment with 5 µM I-BET726. Right: Percentage of cells in subs G1, G1, S, and G2 phases from the cell cycle experiment.

We then analyzed the effect of I-BET726 treatment on MYC-family gene expression in a number of neuroblastoma cell lines. We consistently observed a potent, concentration-dependent decrease in *MYCN* expression, independent of *MYCN* amplification status (Figure 5A). High concentrations of I-BET726 almost completely silenced *MYCN* expression in every cell line tested. In contrast, variable effects were observed on *MYC* expression following I-BET726 treatment. Three out of the four neuroblastoma cell lines with detectable *MYC* expression were examined; one cell line exhibited potent *MYC* suppression, the second showed intermediate effects, and the third was completely insensitive (Figure S12 in File S1). *MYC* down-regulation did not correlate with baseline expression level or sensitivity in the growth-death assay (Table S1, Figure S3 in File S1). Therefore, our observations in neuroblastoma cell lines differ from previous observations in hematologic cancer cell lines, where *MYC* suppression was consistently observed in the context of native, translocated, and amplified loci from a number of different tumor types [11,16–18]. Instead, our data indicate that inhibition of *MYCN* by a BET inhibitor is more ubiquitous and potent than *MYC* in neuroblastoma.

To determine if *MYCN* suppression is due to direct regulation of the *MYCN* locus by BET proteins, we analyzed recruitment of BRD4 to the *MYCN* promoter by chromatin immunoprecipitation (ChIP). BRD4 was specifically enriched at the *MYCN* promoter relative to IgG control ChIP in vehicle-treated samples, but not at an intergenic region on chromosome 12 (Figure 5B). Treatment with I-BET726 diminished BRD4 binding at the *MYCN* promoter in both non-amplified and *MYCN*-amplified cell lines (Figure 5B, 5C), indicating that direct regulation by I-BET726 remains intact upon gene amplification. *MYCN* suppression was further confirmed by Western blot in two *MYCN*-amplified cell lines (Figure 5D). Taken together, these data demonstrate that I-BET726 directly modulates *MYCN* transcription by inhibiting BRD4 recruitment, and represses *MYCN* expression to a high degree regardless of amplification status.

To assess the contribution of *MYCN* suppression to the growth effects observed in *MYCN*-amplified cell lines, we transduced CHP-212 cells with lentiviral constructs for overexpression *GFP* or *MYCN*. *MYCN* expression from the lentiviral vector was not regulated by BET proteins, as treatment with I-BET726 produced no change in N-Myc expression in the *MYCN*-transduced cells, whereas the *GFP*-transduced cells exhibited down-regulation similar to that observed in the parent line (Figure S13A in File S1; Figure 5D). Decreased sensitivity to I-BET726 was observed when *MYCN* could not be silenced (Figure 5E), with a 2.5-fold shift in gIC_50_ in the *MYCN*- versus *GFP*-overexpressing cells. Notably, the concentration of I-BET726 required to observe net cell death was 7-fold higher in *MYCN*-overexpressing cells compared to GFP controls (Figure 5F), suggesting that *MYCN* silencing by I-BET726 contributes more to cytotoxicity than to growth inhibition in this cell line. However, the extent of net cell death at high concentrations of compound were comparable between the two samples (Figure 5F, Figure S13B in File S1), indicating that *MYCN* silencing enhances the cytotoxic response to I-BET726, but is not required to trigger cell death. Cell cycle analysis supports a role for *MYCN* silencing in sensitivity to BET inhibition, as sub-G1 accumulation was only observed in *GFP*-overexpressing cells following treatment with 5 µM I-BET726 (Figure 5G). Similar results were obtained from another *MYCN-*amplified cell line (Figure S14 in File S1), further indicating a contribution of *MYCN* silencing to the potent cytotoxicity observed in these cell lines.

### I-BET726 directly regulates expression of *BCL2*

**Figure 6:**
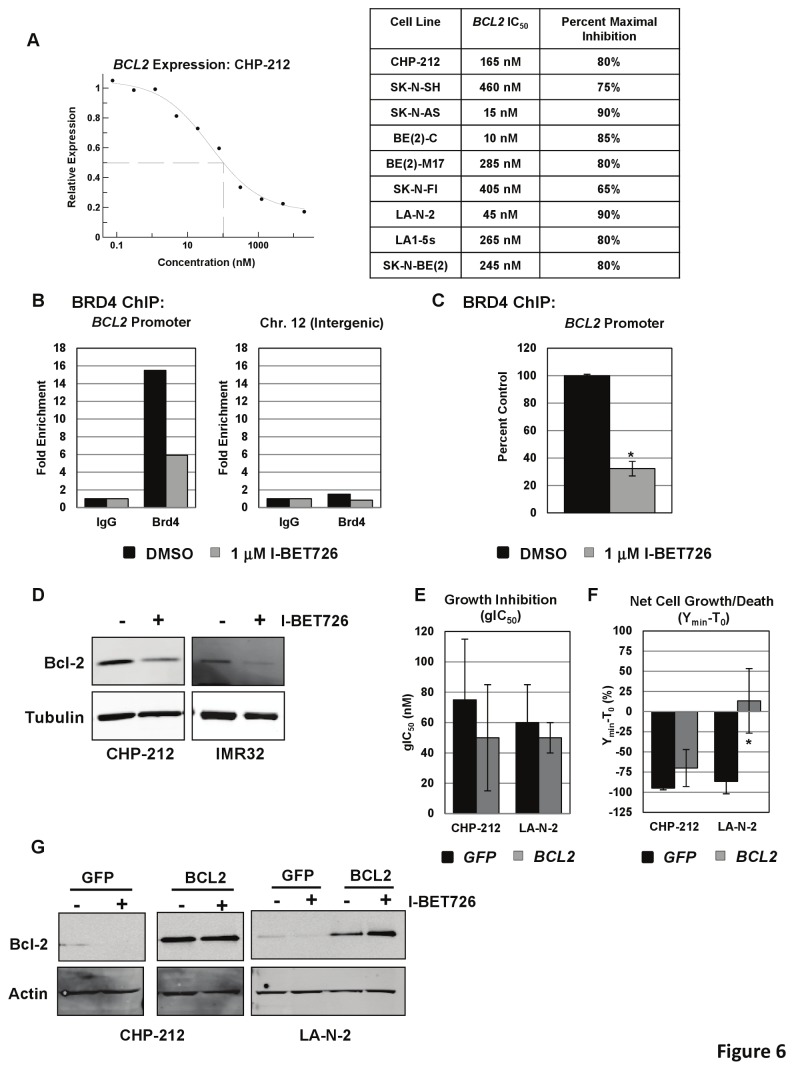
Suppression of *BCL2* expression by I-BET726. (**a**) Left: Concentration response curve for *BCL2* RNA expression following 24 hour treatment with I-BET726 in the CHP-212 cell line. Data was normalized to GAPDH and presented as expression relative to DMSO-treated controls. Data presented as the average of two independent curves from a single experiment, and is representative of data from two independent biological replicates. Right: Table of IC_50_ values and percent inhibition of *BCL2* expression following 24 hour treatment with I-BET726. (**b**) BRD4 ChIP in the non-amplified neuroblastoma cell line SK–N–SH. Binding of BRD4 to the *BCL2* promoter or to an intergenic region on Chromosome 12 following treatment with vehicle or 1 µM I-BET726 for six hours. Data is presented as fold enrichment over signal generated from IgG control immunoprecipitations. Data shown was from a single experiment representative of typical results. (**c**) BRD4 ChIP data at the *BCL2* promoter, presented as percent of vehicle control signal. Data represent the mean value ± standard deviation for three independent biological replicates. Asterisk indicates statistical significance as measured by T-test (p= 0.002). (**d**) Western blot analysis of Bcl-2 expression in the *MYCN*-amplified cell lines CHP-212 and IMR32 following 48 hour treatment with vehicle or 1 µM I-BET726. Tubulin expression included as a loading control. (**e**) gIC_50_ values obtained from CHP-212 or LA-N-2 cells overexpressing *GFP* or *BCL2* following treatment with I-BET726 in a 6 day growth-death assay. Data represents the mean value ± standard deviation from three independent experiments. (**f**) Y_min_-T_0_ values for CHP-212 or LA-N-2 cells overexpressing *GFP* or *BCL2*. Data represents the mean value ± standard deviation from three independent experiments. Asterisk indicates statistical significance as measured by t-test (p= 0.02). (**g**) Western blot analysis of Bcl-2 expression in CHP-212 or LA-N-2 cells overexpressing *GFP* or *BCL2* following 48 hour treatment with DMSO or 1 µM I-BET726. Actin expression included as a loading control.

Among the apoptosis-associated genes identified in the microarray was *BCL2*, an anti-apoptotic gene that is highly expressed in a number of tumor types and associated with *MYCN*-amplification and unfavorable histology in neuroblastoma [30,31]. Consistent with our analysis of MYC family gene expression, we observed no correlation between basal *BCL2* expression and sensitivity to I-BET726 (as measured by gIC_50_ or Y_min_-T_0_) in the neuroblastoma cell line panel (Figure S15 in File S1). We assayed changes in *BCL2* expression following I-BET726 treatment in a number of neuroblastoma cell lines and observed potent and concentration-dependent suppression of *BCL2* in every cell line tested (Figure 6A). *BCL2* down-regulation was previously observed with the BET inhibitor I-BET151 [11], which displaced BET proteins from the *BCL2* transcriptional start site in MLL-fusion leukemia cell lines. Consistent with these observations, we detected BRD4 enrichment at the *BCL2* promoter in the SK–N–SH cell line (Figure 6B), and treatment with I-BET726 abrogated this interaction (Figure 6B, 6C). Analysis of two additional neuroblastoma cell lines revealed reduced Bcl-2 protein levels upon treatment with I-BET726 (Figure 6D), confirming the critical role of BET proteins in maintaining *BCL2* expression.

We then assessed the contribution of *BCL2* silencing to I-BET726-induced cytotoxicity by analyzing responses in CHP-212 and LA-N-2 cells transduced with lentiviral expression vectors for *GFP* or *BCL2*. gIC_50_ values were comparable between *GFP*- and *BCL2*-overexpressing cells from each cell line (Figure 6E), indicating that *BCL2* silencing has no effect on growth inhibition in either cell line. Effects on cytotoxicity were variable. Minimal changes in sensitivity, net cell death, or caspase induction were observed in CHP-212 upon over-expression of *BCL2* (Figure 6F; Figure S16A, S16C in File S1). In contrast, a complete loss of net cell death and caspase induction was observed in LA-N-2 (Figure 6F; Figure S16B, S16D in File S1). Baseline levels of Bcl-2 protein expression in *BCL2*-transduced lines were much higher than in the GFP controls (Figure 6G); as a result, the potency shifts observed in the *BCL2*-overexpressing lines may over-estimate the contribution of *BCL2* silencing to responses observed in the parent cell lines. Nonetheless, it is clear that the relative contribution of *BCL2* silencing to I-BET726 cytotoxicity in neuroblastoma cell lines is variable and, in some cases, has little effect on potency.

### I-BET726 inhibits neuroblastoma tumor growth

**Figure 7:**
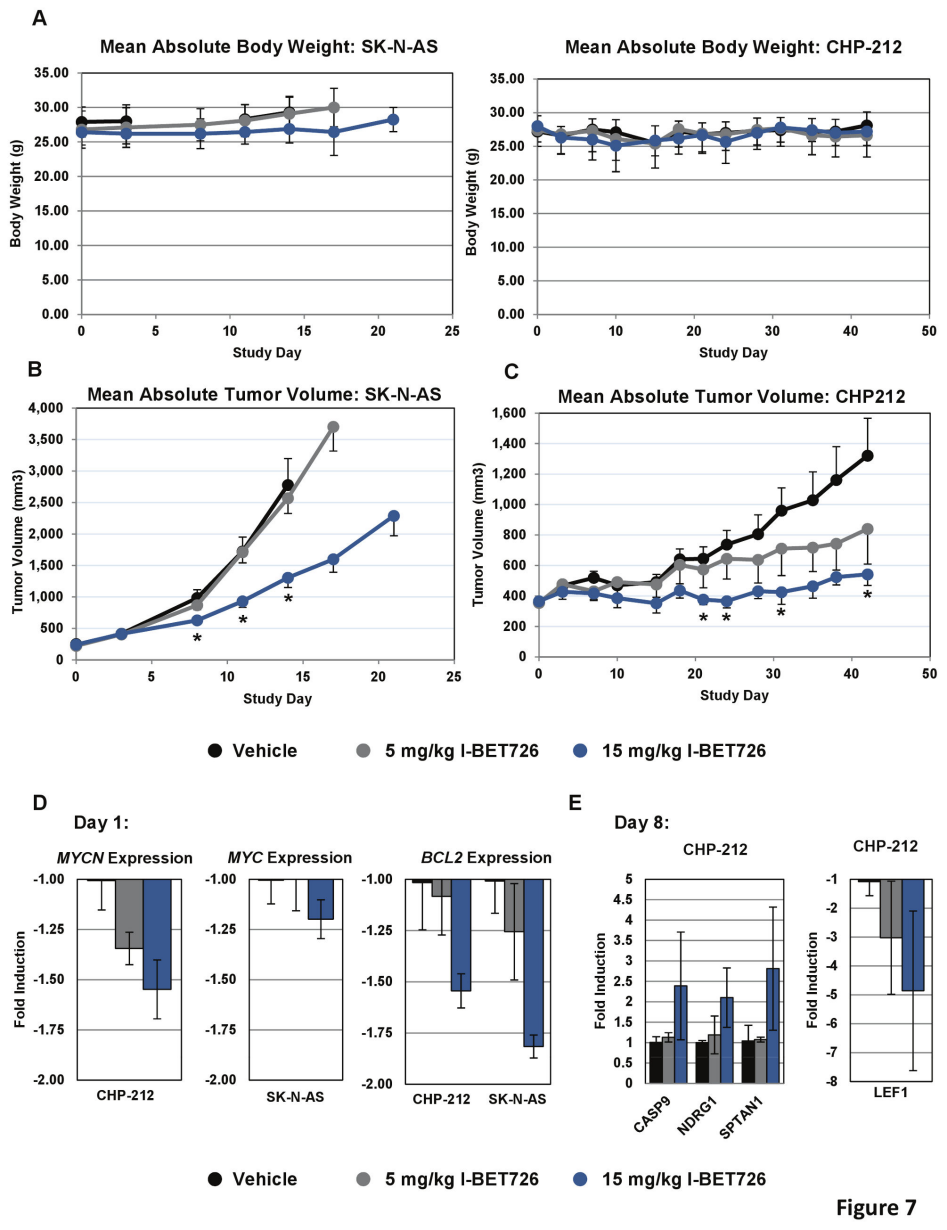
Analysis of I-BET726 activity *in vivo*. (**a**) Mean absolute body weight ± SD for mice in the SK–N-AS (left) and CHP-212 (right) xenograft studies treated with vehicle, 5 mg/kg, or 15 mg/kg I-BET726. (**b**) Mean absolute tumor volumes ± SEM for SK–N-AS subcutaneous xenografts following treatment with 5 mg/kg or 15 mg/kg I-BET726. Asterisks indicate statistical significance as measured by t-test (p <0.05). Tumor growth inhibition (TGI) for the 15 mg/kg group was 58% on day 14 (n= 9; p= 0.0060). (**c**) Mean absolute tumor volumes ± SEM for CHP-212 subcutaneous xenografts following treatment with 5 mg/kg or 15 mg/kg I-BET726. Asterisks indicate statistical significance as measured by T-test (p <0.05). TGI for 5 mg/kg was 50% on Day 42 (n= 8; p= 0.1816). TGI for 15 mg/kg was 82% on Day 42 (n=5; p =0.0488). (**d**) Pharmacodynamic analysis in CHP-212 and SK–N-AS xenografts 8 hours after initial dose of I-BET726. qRT-PCR analysis of *MYCN*, *MYC*, and *BCL2* expression following I-BET726 treatment in the indicated models. Data is presented as fold induction compared to vehicle treated controls, and represents the average ± SD of data from three animals. (**e**) qRT-PCR analysis of apoptotic pathway and N-Myc pathway genes in CHP-212 xenografts 8 hours following treatment with I-BET726 on day 8 of study. Data is presented as described in (d).

To examine the therapeutic potential of I-BET726 *in vivo*, we established subcutaneous xenograft models of non-*MYCN*-amplified and *MYCN*-amplified neuroblastoma in immunocompromised mice using the SK–N-AS and CHP-212 cell lines, respectively. I-BET726 was administered by oral gavage once daily at doses of 5 mg/kg or 15 mg/kg. Blood and tumor concentrations of I-BET726 were comparable between the two models, confirming that a similar exposure was achieved in the two studies (Figure S17 in File S1). The 5 mg/kg dose was well-tolerated in both studies (Figure 7A). Body weight loss was observed in some mice in the 15 mg/kg group in both studies following repeated exposure to the compound, and these mice were euthanized if body weight loss exceeded 20%. Remaining mice in the 15 mg/kg groups exhibited stable body weight throughout the duration of the studies (Figure 7A), and exhibited no gross toxicities associated with compound treatment.

In the SK–N-AS model, mice in the vehicle group were euthanized on day 14 due to large tumor size (Figure 7B). While there was no significant difference in tumor growth between the vehicle and 5 mg/kg group, we observed 58% tumor growth inhibition (TGI) in the 15 mg/kg group on day 14 of the study (n=9; p= 0.0060). Mice in the 15 mg/kg group were treated for an additional 7 days before tumor volume reached a level comparable to that observed in the vehicle group, at which point the study was terminated. Tumors in the CHP-212 model grew much more slowly. After 42 days, tumors in vehicle-treated mice were only half the size those in the SK–N-AS model at the end of the study (Day 14). In the CHP-212 model, treatment with 5 mg/kg I-BET726 resulted in TGI equal to 50% (n=8; p= 0.1816; Figure 7C), and mice in the 15 mg/kg group exhibited a TGI of 82% at the end of the study (n=5; p =0.0488). The enhanced sensitivity to I-BET726 at the lower dose in the CHP-212 model is consistent with our *in vitro* growth data; however caution must be taken when comparing these two models due to both the differences in tumor growth rate and the reduced number of animals in the 15 mg/kg dosing groups. Nonetheless, our data confirms the sensitivity to I-BET726 observed in these neuroblastoma cell lines *in vitro*.

Pharmacodynamic analyses were performed 8 hours following the initial dose of I-BET726 in each study. Consistent with our *in vitro* studies, we observed a dose-dependent decrease in *MYCN* and *BCL2* expression in the xenograft models following treatment with I-BET726 (Figure 7D). SK–N-AS cells express *MYC* as opposed to *MYCN*, and we detected no significant decrease in *MYC* expression in this cell line. Additional analysis of gene expression changes following eight days of dosing in CHP-212 indicated that similar pathways were affected *in vitro* and *in vivo*, as we observed expression changes for genes involved in apoptosis as well as N-Myc regulated genes (Figure 7E). Taken together, these data highlight the potential of BET inhibitors such as I-BET726 as potent anti-tumor agents in neuroblastoma, in part through the alteration of apoptotic and N-Myc-driven pathways.

## Discussion

Previous studies reported enhanced sensitivity to BET inhibitors in hematologic cancer models with high *MYC* expression due to potent *MYC* silencing and down-regulation of Myc-driven transcriptional programs [16–18]. Apart from NMC, there are few reports to date on the activity of BET inhibitors in solid tumors, and it remains unclear whether a similar link may exist between MYC family gene suppression and sensitivity in solid tumor models. Here we show that a novel BET inhibitor, I-BET726, exhibits potent anti-proliferative activity in models of neuroblastoma, a solid tumor associated with a high frequency of *MYCN* gene amplifications. Our *in vitro* data suggests that BET inhibition triggers a potent cytotoxic response in neuroblastoma cell lines irrespective of *MYCN* copy number. Similarly, I-BET726 anti-tumor activity was observed in both non-*MYCN-*amplified and *MYCN-*amplified neuroblastoma models. *MYCN* amplification occurs in approximately 20% of primary neuroblastoma tumors and strongly correlates with advanced stage disease and resistance to therapy [32]. Our data suggest that BET inhibitors such as I-BET726 could serve as novel therapeutic agents for neuroblastoma, benefitting patients with even the most aggressive forms of the disease.

Surprisingly, *MYC* regulation and cellular response to BET inhibition differ between hematologic and solid tumor models such as neuroblastoma, as *MYC* suppression was variable in *MYC*-expressing neuroblastoma cell lines and did not positively correlate with sensitivity to I-BET726. These findings are consistent with recent reports on BET inhibitor activity in lung adenocarcinoma and glioblastoma [33,34], and suggest that the role of BET proteins in regulating *MYC* expression may be tumor type- or cell line-specific.

In contrast to *MYC*, potent *MYCN* suppression was observed in every neuroblastoma cell line tested that expressed *MYCN*. Importantly, dose-dependent inhibition of *MYCN* expression was also observed in the CHP-212 xenograft model, indicating that N-Myc driven pathways are similarly affected *in vitro* and *in vivo*. The growth effects observed following I-BET726 treatment were consistent with a loss of N-Myc activity, as targeted inhibition of *MYCN* expression was shown to trigger growth inhibition, differentiation, and apoptosis in neuroblastoma cell lines [35,36]. ChIP analysis confirmed recruitment of BRD4 to the *MYCN* promoter. The *MYCN* gene was also reported to be activated by E2F proteins [37]; given the role of BRD2 in recruiting these proteins to chromatin, it is possible that BRD2 also plays a critical role in *MYCN* expression.

Consistent with I-BET726-mediated *MYCN* suppression, we observed changes in expression of a number of known MYC-family downstream targets in the microarray study. Recently, a *MYCN* signature was proposed in neuroblastoma based upon expression changes following knockdown of *MYCN* in the *MYCN*-amplified IMR32 cell line that also correlated with *MYCN* expression in neuroblastoma tumors [38]. Of the 157 genes identified in this signature, 35 were found to be significantly regulated by I-BET726 in the CHP-212 microarray study, with the majority (26 of 35) exhibiting changes consistent with *MYCN* suppression. The 9 remaining overlapping genes include 8 that are normally silenced by *MYCN* suppression, but are also silenced by I-BET726 treatment, perhaps reflecting direct silencing of these genes by BET inhibition that supersedes any indirect effects of *MYCN* silencing. Taken together, these data suggest that I-BET726 modulates expression of genes potentially relevant for N-Myc driven tumor biology in primary neuroblastomas. Prolonged exposure to I-BET726 might reveal additional overlap between the two data sets, since our microarray study was not optimized to capture indirect expression changes associated with silencing of *MYCN*.

Concentration-dependent expression changes were confirmed by qRT-PCR for several N-Myc target genes including *NME2*, an inhibitor of differentiation located on a region of chromosome 17q that is prone to amplification in aggressive neuroblastomas [32,39]. 17q amplification is often associated with *MYCN*-amplification, resulting in over-expression of *NME2* both through increased copy number and increased N-Myc-mediated transcription. Two additional genes on this region of 17q thought to promote the aggressive phenotype, *NME1* and *BIRC5* (survivin), were also down-regulated by I-BET726 in the microarray. It is currently unclear whether these effects are due to direct inhibition of BET proteins at these loci, indirect effects of silencing *MYCN* expression, or a combination of the two mechanisms. Understanding the mechanism of suppression at these genes will have important implications with respect to I-BET726 activity in neuroblastoma, as direct inhibition of these genes could potentially reverse the malignant phenotype of 17q alterations even in the absence of *MYCN* amplification.

Our observations of potent BET inhibitor activity in neuroblastoma are consistent with a recent report using a different BET inhibitor, JQ1, which was similarly shown to inhibit expression of *MYCN* and downstream N-Myc target genes [40]. Our data support a role for *MYCN* silencing in sensitivity to I-BET726, particularly with respect to cytotoxicity. However, the lack of complete rescue from cell death upon ectopic *MYCN* over-expression indicates that perturbations in other pathways play a role in the cellular response as well. We and others have observed *BCL2* silencing upon treatment with BET inhibitors, accompanied by a loss of BRD4 binding at the *BCL2* locus (Figure 6) [11]. Knockdown of *BCL2* in neuroblastoma cell lines triggers apoptosis [41], and ectopic overexpression of *BCL2* inhibits apoptosis [42,43]. Bcl-2 is highly expressed in a significant percentage of primary neuroblastoma tumors (16-52%), and expression correlates with markers of poor prognosis including *MYCN*-amplification [31,41,44]. Co-expression of *BCL2* and *MYCN* in neuroblastoma cell lines increases tumorigenicity and protects cells from apoptosis [45]. Thus, silencing of *BCL2* via BET inhibition may contribute to the potent cytotoxic responses observed in some *MYCN*-amplified cell lines, and perhaps be a primary driver of cytotoxicity in other cell lines, such as SH-SY5Y, that lack *MYCN*-amplification but express high levels of *BCL2* [30].

It is important to note, however, that *BCL2* silencing did not predict cytotoxicity, as potent *BCL2* suppression was observed in every cell line tested regardless of response (Figure 6A). Additionally, loss of *BCL2* silencing resulted in dramatically different effects on cytotoxicity following I-BET726 treatment in the two cell lines analyzed (Figure 6E, F). Cytotoxicity in response to I-BET726 may depend on a specific cell line’s dependence on *BCL2* for survival, as reflected in the expression levels of other important pro- and anti-apoptotic factors. A large number of expression changes were observed in the microarray for genes involved in apoptosis; additional investigation will be required to fully understand how these changes in expression translate into cellular response to I-BET726.

GSEA revealed a significant enrichment of several terms associated with E2F binding motifs in the SK–N–SH cell line, but only one in CHP-212 (Table S4). E2F signatures were previously observed to be significantly down-regulated upon treatment with another BET inhibitor in multiple myeloma cell lines containing various activating genetic lesions at the *MYC* locus [18]. Additionally, BRD2 has been shown to interact with E2F family members and regulates the expression of several E2F-dependent cell cycle genes [6,7]. It is currently unclear whether the difference in regulation of E2F signatures between SK–N–SH and CHP-212 is linked to their *MYCN-*amplification status, or whether it may simply result from differences in basal expression or activity of BRD2 or E2F1 in these cell lines. Analyses in additional cell lines possessing or lacking *MYCN* amplification is warranted to further characterize the relationship between *MYCN* status and changes in these E2F signatures.

The potent effects observed on *BCL2* and *MYCN* expression in our study highlight the potential therapeutic value of BET inhibitors in other solid tumors driven by high expression of these oncogenes, including small cell lung cancer, medulloblastoma, retinoblastoma, and rhabdomyosarcoma [22,46]. Given the potent activity already observed in neuroblastoma and Myc-driven hematologic cancers, characterization of BET inhibitors in these additional tumor types is warranted.

In summary, we have identified neuroblastoma as a solid tumor model highly sensitive to BET protein inhibition. Taken together with previous observations of anti-tumor activity in NMC, lung adenocarcinoma, glioblastoma, and a number of hematologic malignancies [11,12,16–18,33,34], our study highlights the potential of BET inhibitors like I-BET726 as effective therapeutic agents in a wide variety of cancers. Further investigation of the transcriptional programs regulated by BET family proteins likely will uncover additional mechanisms through which BET inhibitors can offer therapeutic benefit in cancer and other diseases.

## Materials and Methods

### Cell lines and Reagents

Cell lines were obtained from ATCC (Manassas, VA) or Sigma-Aldrich (St. Louis, MO) and were grown in RPMI-1640 medium containing 10% FBS, 2 mM GlutaMAX (Life Technologies, Grand Island, NY), and 1 mM sodium pyruvate. Antibodies and qPCR primers are listed in Methods S1.

### I-BET726 Characterization

I-BET726 synthesis is described in International Patent Number WO 2011/054848 A1. Determination of crystal structure, binding affinity to BET proteins, and selectivity was performed as described [10,11,13], and in Methods S1.

### Cell Line Growth-Death Assay

Cell line growth-death assays were performed as described [47], with a few modifications. Briefly, cells were seeded into 384-well or 96-well plates at a density optimized for 6 days of growth. The following day, T_0_ measurements were taken using CellTiter-Glo (CTG; Promega, Madison, WI), CellTiter-Fluor (Promega), or CyQuant Direct (Life Technologies), following the manufacturer’s instructions. Plates were read on an Envision (PerkinElmer, Waltham, MA), Safire 2 (Tecan, Durham, NC), or SpectraMax Gemini EM (Molecular Devices, Sunnyvale, CA) plate reader. Remaining plates were treated with DMSO or a titration of I-BET726. Cells were incubated for 6 days and developed as described above. Results were plotted as a percentage of the T_0_ value, normalized to 100%, versus concentration of compound. A 4-parameter equation was used to generate concentration response curves. Growth IC_50_ (gIC_50_) values were calculated at the mid-point of the growth window (between DMSO and T_0_ values). Y_min_-T_0_ values were calculated by subtracting the T_0_ value (100%) from the Y_min_ value on the curve, and are a measure of net population cell growth or death.

### Cell Cycle Analysis

Samples were prepared as described [48] and analyzed on a FACSCalibur or LSR II flow cytometer (BD Biosciences, Franklin Lakes, NJ). Histograms were generated and cell cycle analysis was performed using FlowJo software (Tree Star, Inc., Ashland, OR).

### Caspase 3/7 Assay

Cell plating and dosing were performed as described above for the growth-death assay. CTG and Caspase-Glo 3/7 (Promega) readings were taken following the procedure described above for CTG. Caspase-Glo 3/7 readings were normalized to CTG readings from the same treatment group to correct for differences in cell number. Results were plotted as fold-induction relative to DMSO-treated samples from the corresponding time point.

### Illumina Microarray Profiling

Three biological replicates of CHP-212 or SK–N–SH cells were treated with DMSO, 100 nM I-BET726, or 1 µM I-BET726 for 16 hours. RNA isolation and gene expression profiling were performed at Expression Analysis (Durham, NC). Briefly, total RNA was isolated, examined with an Agilent BioAnalyzer for integrity and yield, labeled, and hybridized onto the Illumina Human HT–12 v4 Expression BeadChip according to the manufacturer’s instructions (Illumina, San Diego, CA). Signal values were normalized and log_2_ transformed. Differential analysis of probes was performed by fitting data to linear models and performing pair-wise contrasts of interest using a moderated t-statistic. p-values generated were adjusted for multiple testing by applying Benjamin Hochberg’s FDR correction. Significant probes were filtered for detection (p<0.05 in at least sample), average fold change >2 or <-2 and FDR <0.1. Statistical analyses were performed using the limma package from Bioconductor (http://www.bioconductor.org/). Hierarchical clustering was performed on significant gene lists using complete linkage and Pearson correlation parameters. Functional analyses of these lists in terms of Gene Ontology Biological Process (GO BP) or pathway enrichment were performed at a gene-level using DAVID (http://david.abcc.ncifcrf.gov/). Gene Set Enrichment Analysis was performed using GenePattern [49,50]. Gene set permutations were used to identify significantly enriched gene sets from the c2 (curated gene sets) and c3 (motif gene sets) MSigDB collections using the signal-to-noise metric for vehicle versus 1µM I-BET726-treated samples.

The microarray dataset has been deposited in GEO under the accession number #GSE47386.

### MYCN Transcriptional Regulation Network Analysis

A neighborhood network of *MYCN* interactions was constructed using MetaCore (Thomson Reuters; http://portal.genego.com). The network was built using the “Expand by one interaction” algorithm for downstream network objects and filtered for transcriptional regulation and binding interactions. Genes with corresponding differential expression from our microarray dataset were highlighted.

### qRT-PCR Analysis

RNA was purified using the TurboCapture 96 kit or RNEasy Mini kit (Qiagen, Valencia, CA) and cDNA was generated using the High Capacity cDNA Reverse Transcription kit (Life Technologies), following the manufacturers’ instructions. TaqMan analysis was performed on an Applied Biosystems ViiA7 real-time PCR machine, using *GAPDH* or *HPRT* as internal controls. Relative expression compared to DMSO was calculated using the 2^-ΔΔCt^ method. Concentration response curves were generated with a 4-parameter model using XLfit software (IDBS, Alameda, CA). Additional details for RNA expression analysis can be found in Methods S1.

### Chromatin Immunoprecipitation (ChIP)

ChIP experiments were performed as described [51]. Detailed methods for ChIP are included in Methods S1.

### Western Blot Analysis

Lysates were generated in Cell Lysis buffer (Cell Signaling Technology, Danvers, MA) containing 1x protease and phosphatase inhibitor cocktail (Cell Signaling Technology), following the manufacturer’s protocol. Protein concentration was determined via BCA protein assay (Thermo Scientific, Rockford, IL), using BSA as a standard. Equivalent amounts of protein were separated by SDS-PAGE and transferred onto nitrocellulose membranes. Antibodies were diluted in Odyssey blocking buffer (LI-COR Biosciences, Lincoln, NE) containing 0.05% Tween-20 at the manufacturer’s recommended dilutions. Images were obtained on an Odyssey infrared imaging system (LI-COR Biosciences).

### In vivo Studies

All studies were conducted after review by the Institutional Animal Care and Use Committee at GSK and in accordance with the GSK Policy on the Care, Welfare and Treatment of Laboratory Animals. The Institutional Animal Care and Use Committee at GSK specifically approved these studies. CHP-212 (1x10^7^) or SK–N-AS (5x10^6^) cells in 100% matrigel (BD Biosciences) were implanted subcutaneously into the right flank of approximately 9 week old female nude (Crl:CD-1-Foxn1 nu) mice. Tumors were measured with calipers and randomized using stratified sampling according to tumor size into treatment groups of 10 mice. I-BET726 formulated as a spray dried dispersion was prepared as a suspension in 1% methylcellulose vehicle. I-BET726 in vehicle or vehicle alone was administered orally by individual body weight at 10mls/kg. Mice were weighed and tumors were measured with calipers twice weekly, and mice were observed daily for any adverse treatment affects. Mice were euthanized using CO_2_ inhalation according to AVMA guidelines after two consecutive tumor measurements greater than 2500mm^3^, or if body weight loss greater than 20% was observed. For mouse pharmacodynamic studies, mice were euthanized as described above. Tumors were harvested from euthanized mice and placed in RNAlater (Life Technologies) for RNA isolation as described in Methods S1. Blood was collected after euthanasia via cardiac puncture.

## Supporting Information

File S1 Contains Figures S1-S17.
(PDF)

Table S1 **Neuroblastoma Growth-Death Analysis.**
(XLS)

Table S2 **Differentially Expressed Gene Lists for CHP-212 and SK–N–SH.**
(XLS)

Table S3 **Pathway Enrichment in CHP-212 and SK–N–SH.**
(XLS)

Table S4 **GSEA in CHP-212 and SK–N–SH.**
(XLS)

Table S5 ***MYCN* Network Genes in CHP-212 and SK–N–SH.**
(XLSX)

Methods S1 (DOCX)
