# Supplementary material for: BET Inhibition Silences Expression of MYCN and BCL2 and Induces Cytotoxicity in Neuroblastoma Tumor Models
Source: PLoS One. 2013 Aug 23;8(8):e72967. doi: 10.1371/journal.pone.0072967 (PMC3751846; doi:10.1371/journal.pone.0072967)
Supplement: File S1 — Contains Figures S1-S17. (PDF) [file pone.0072967.s001.pdf]

BRD4:

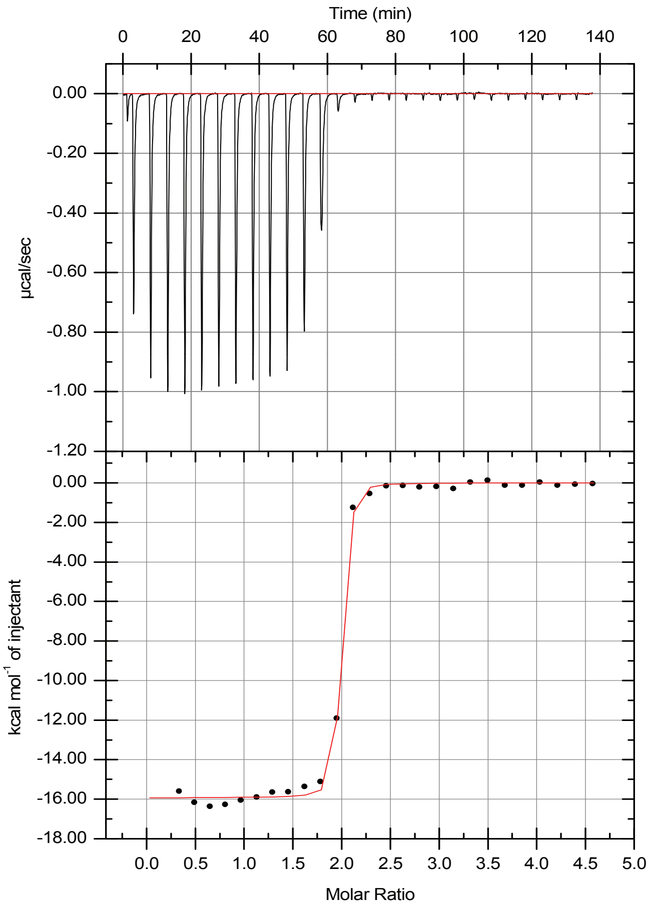

CREBBP:

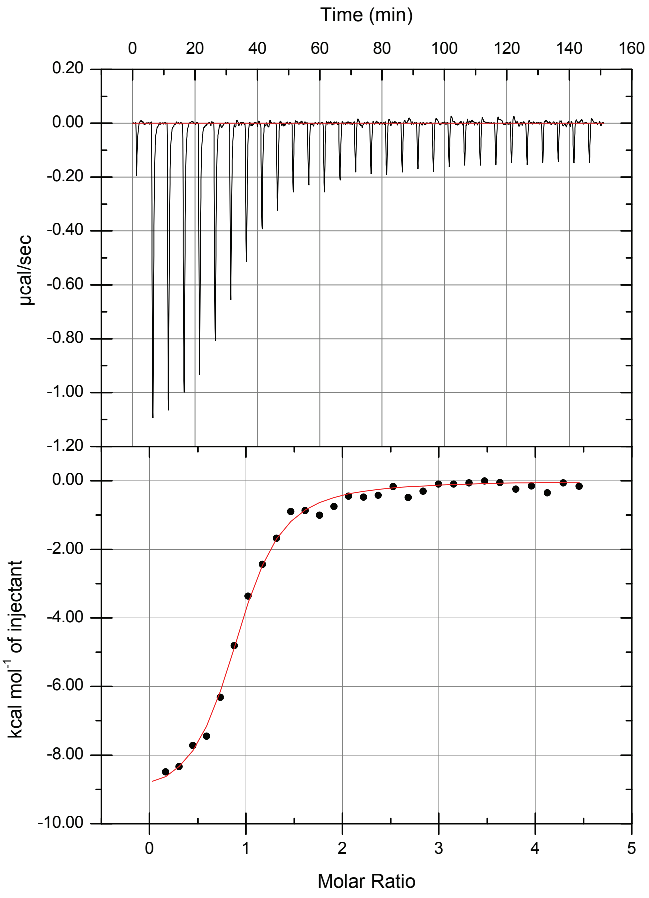

Protein	K_D (nM)	ΔH (kcal/mol)	ΔS (cal/mol/deg)	N (stoichiometry ligand:protein)
BRD4	4.4±0.9	-15.9±0.08	-15.2	1.9 :1
CREBBP	6300±500	-4.35±0.08	9.2	1.0: 1

Figure S1. Determination of binding affinity of BRD4 (left) and CREBBP (right) to I-BET726 by isothermal calorimetry. Summary of ITC parameters including calculated K_D values are indicated for each protein.

Figure S1

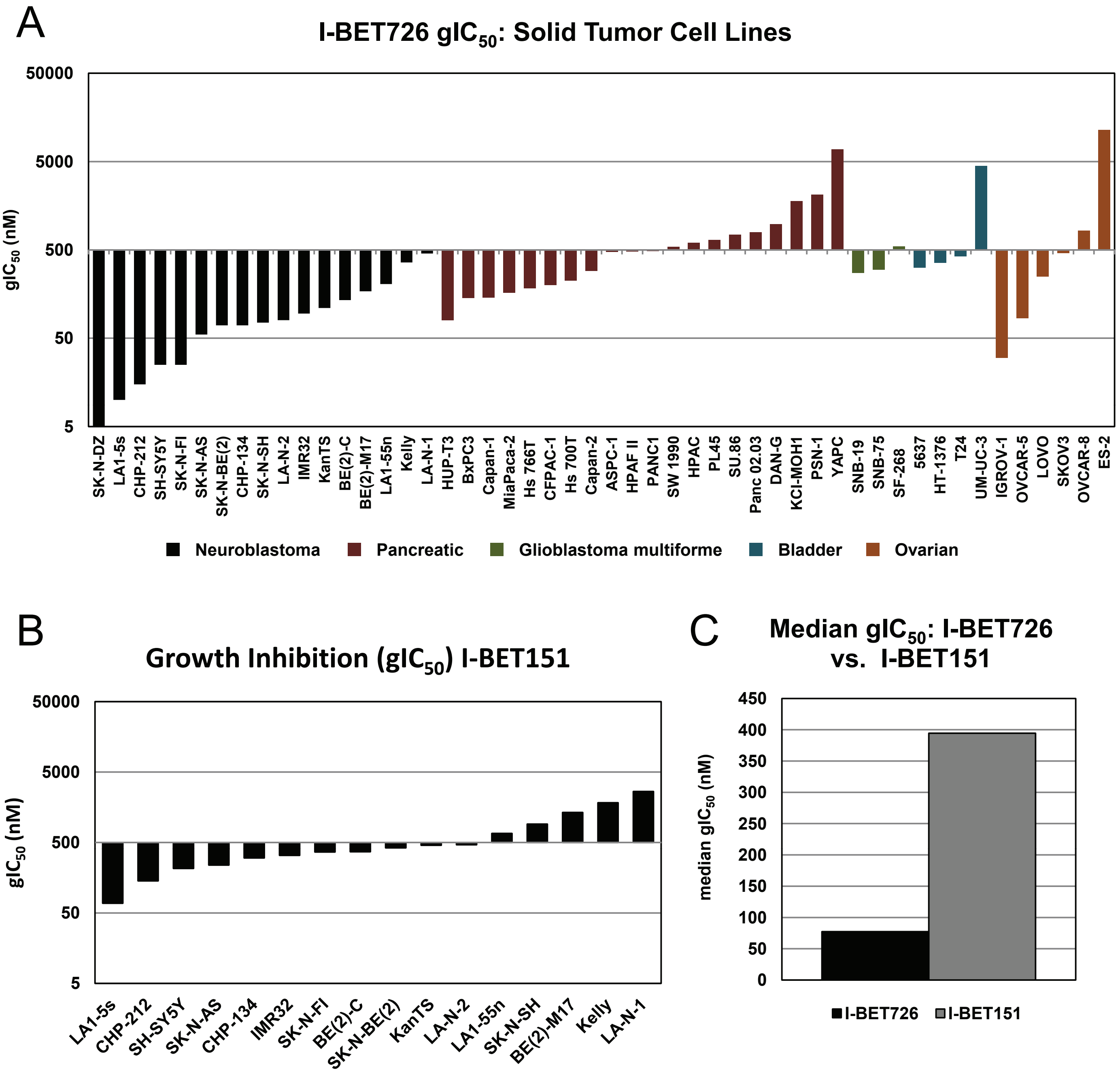

Figure S2. (a) Analysis of I-BET726 activity in a panel of solid tumor cell lines. Growth IC_{50} values in the indicated cell lines following treatment with a titration of I-BET726 in a 6 day growth-death assay. **(b)** Growth IC_{50} values for neuroblastoma cell lines treated with I-BET151 for 6 days. **(c)** Median gIC_{50}s for I-BET726 and I-BET151 in the 6 day growth-death assay.

Figure S2

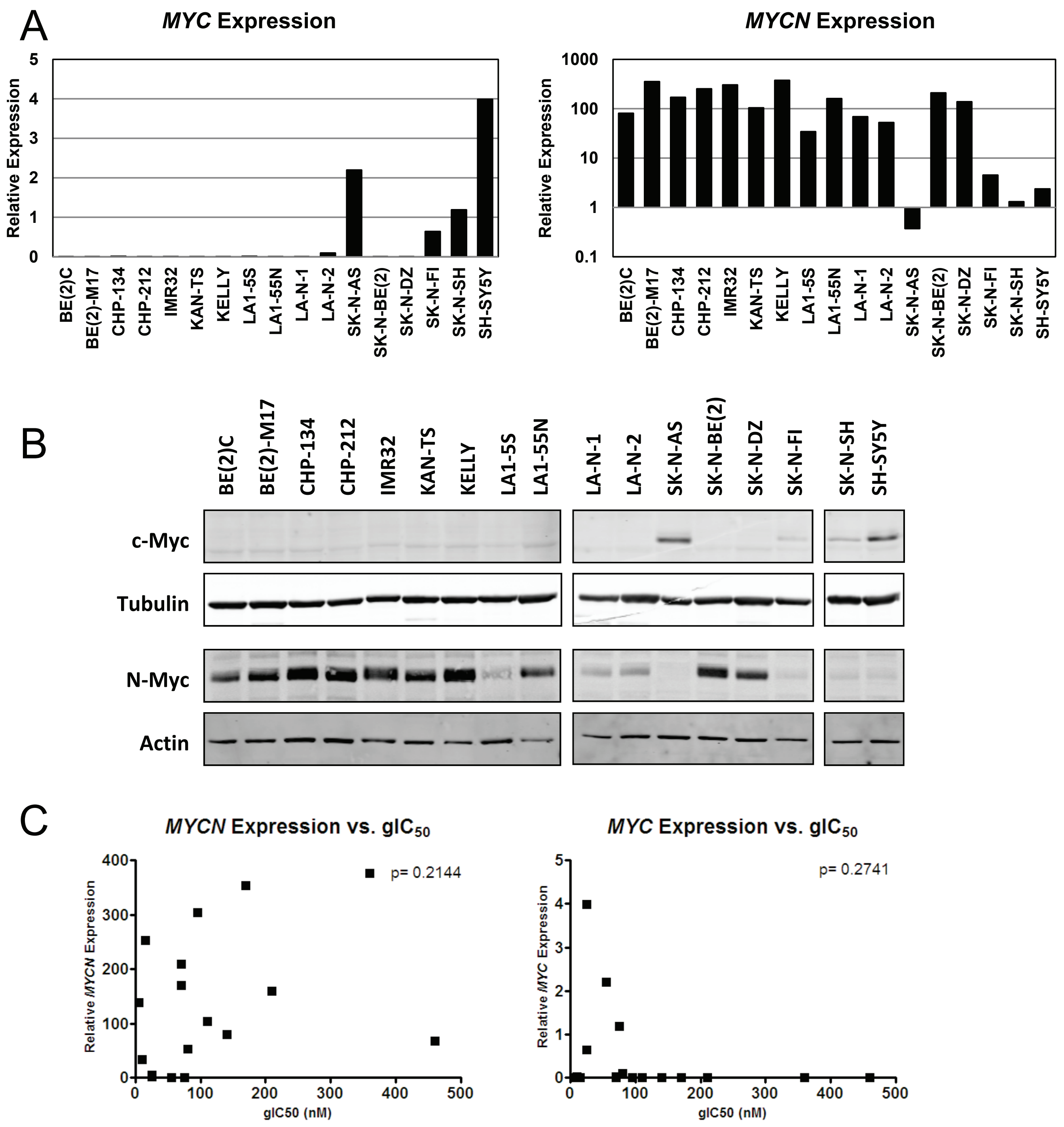

Figure S3. Analysis of *MYC and MYCN* expression in a panel of neuroblastoma cell lines. (**a**) qPCR determination of *MYC and MYCN* RNA expression in the indicated cell lines. Data were normalized to expression of *HPRT*, and are presented as relative expression compared to a universal reference RNA sample generated from a collection of different human tissues (Clontech). (**b**) Western blot analysis of c-Myc and N-Myc expression in the indicated cell lines. **(c)** Correlation of I-BET726 sensitivity as measured by gIC_{50} to relative RNA expression of *MYCN* (left) or *MYC* (right) in the neuroblastoma cell line panel. p-values from two-tailed Pearson correlation analyses are indicated for each comparison.

Figure S3

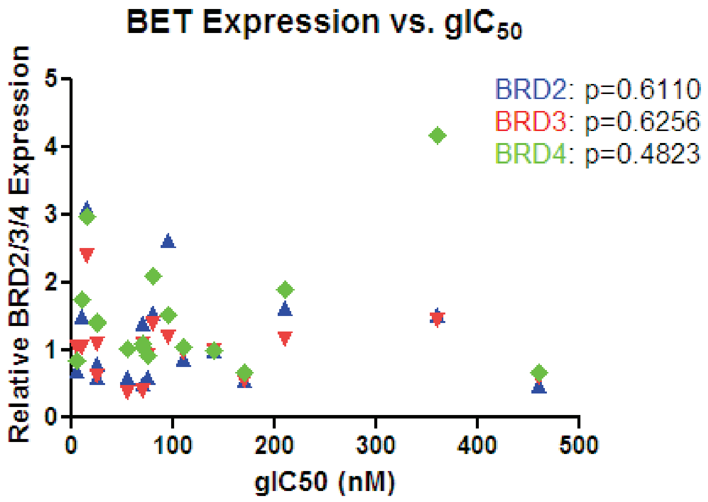

Figure S4. Correlation of I-BET726 sensitivity as measured by gIC_{50} to relative RNA expression of BRD2, BRD3, or BRD4 in the neuroblastoma cell line panel as described in Figure S3C. p-values are indicated for each comparison.

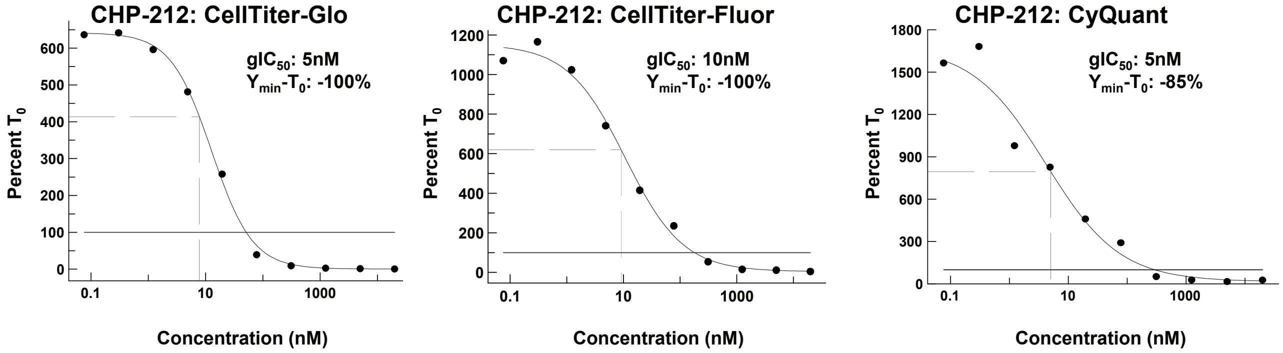

	CellTiter-Fluor		CyQuant	
**Cell Line**	**gIC_{50}**	**Y_{min}-T_0**	**gIC_{50}**	**Y_{min}-T_0**
**CHP-212**	**30 nM**	**-100%**	**10 nM**	**-85%**
**LA1-5s**	**25 nM**	**-80%**	**10 nM**	**-95%**
**SK-N-BE(2)**	**20 nM**	**-100%**	**80 nM**	**-90%**
**SK-N-AS**	**130 nM**	**90%**	**135 nM**	**25%**

Figure S5. Analysis of effects of I-BET726 on cell growth and death using CellTiter-Fluor and CyQuant assays. Top: growth curves generated for I-BET726 in the CHP-212 cell line using CellTiter-Glo, CellTiter-Fluor, and CyQuant assays. gIC_{50} and Y_{min}-T_0 values for each assay are indicated. Bottom: Average gIC_{50} and Y_{min}-T_0 values determined for I-BET726 in the indicated cell lines in the CellTiter-Fluor and CyQuant assays.

Figure S5

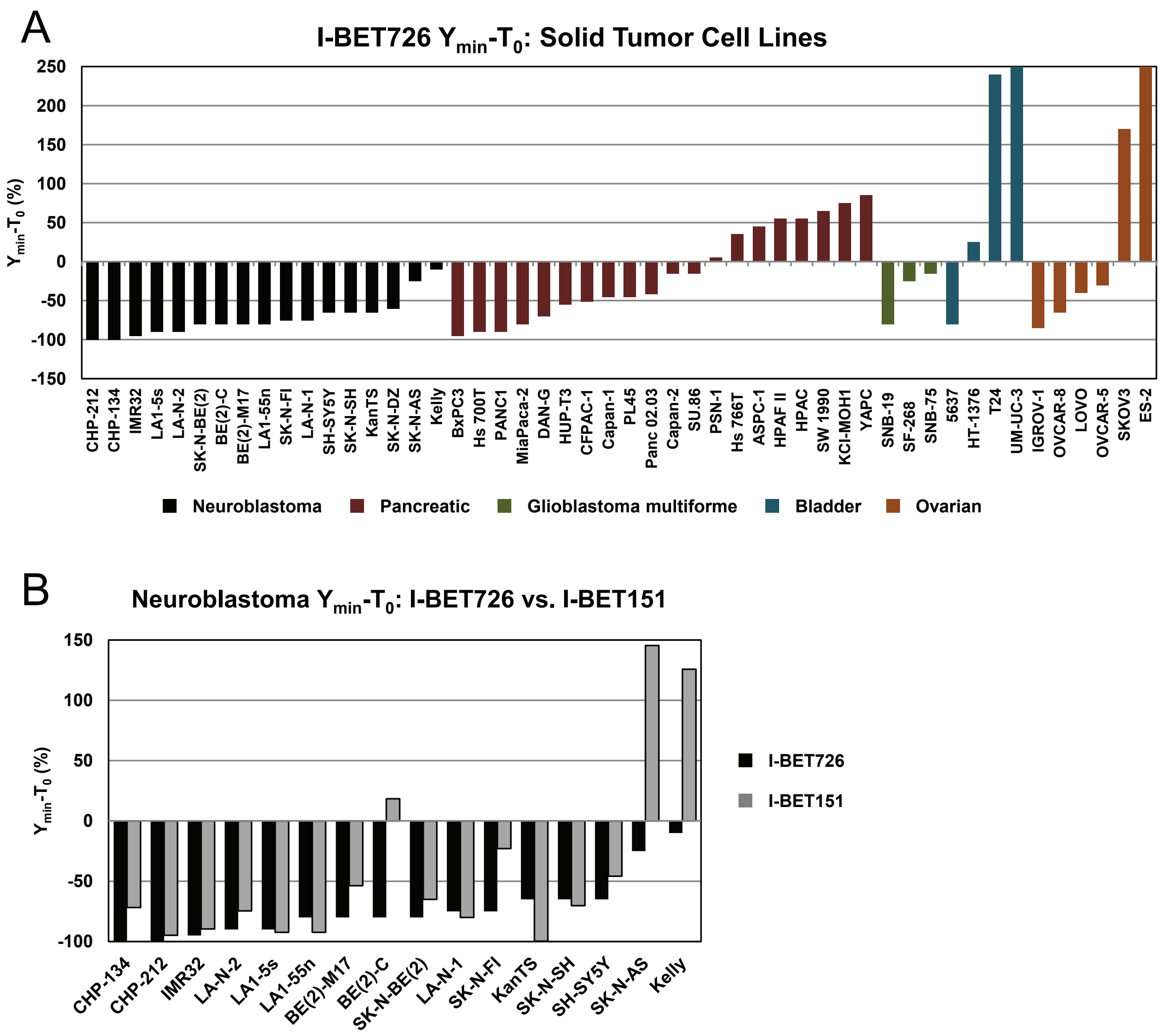

Figure S6. Analysis of BET inhibitor-induced cytotoxicity in a panel of solid tumor cell lines. **(a)** Y_{min}-T_0 values in the indicated cell lines following treatment with a titration of I-BET726 in a 6 day growth-death assay. **(b)** Comparison of Y_{min}-T_0 values obtained in neuroblastoma cell lines following 6 days treatment with either I-BET726 (black bars) or I-BET151 (gray bars).

Figure S6

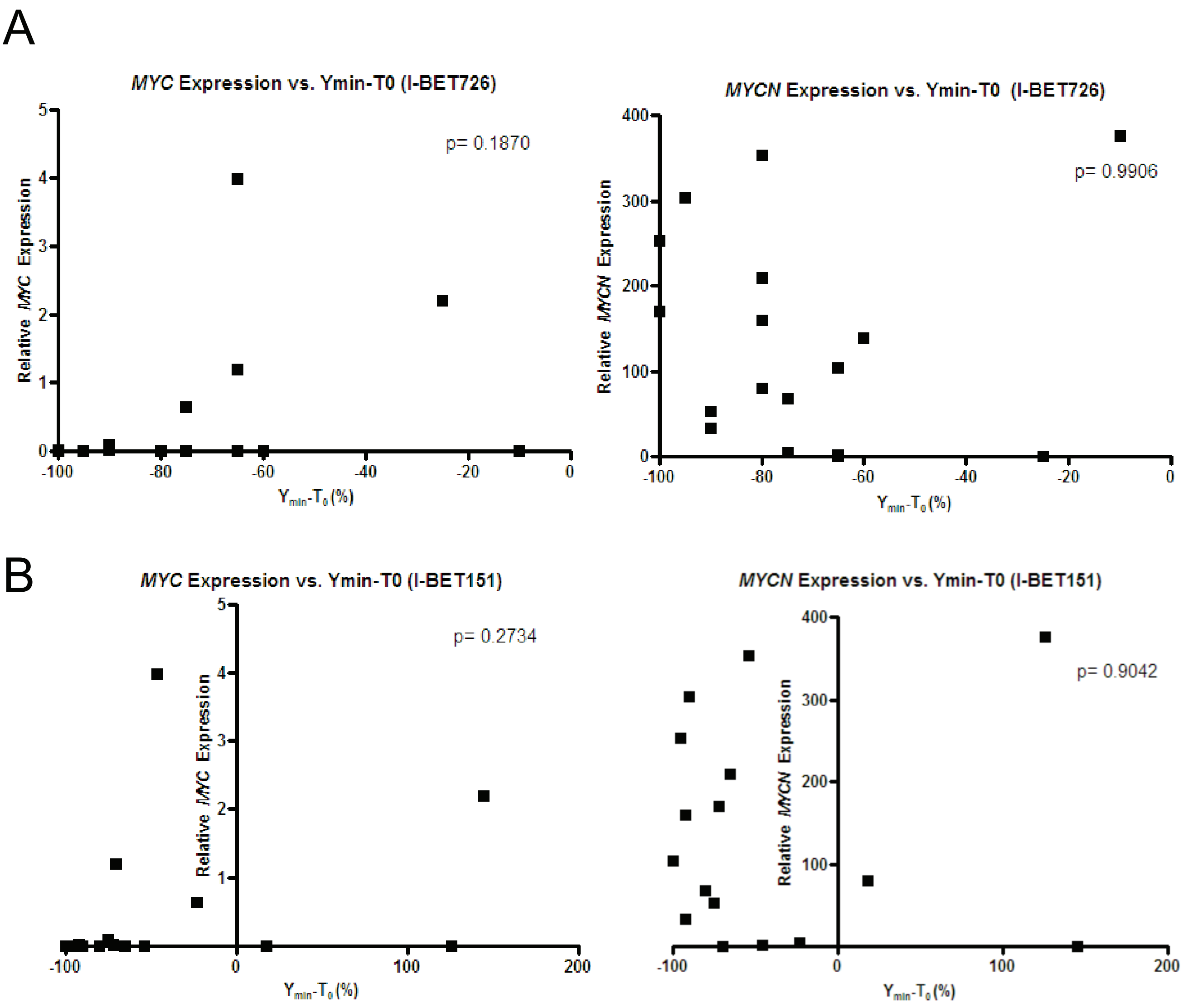

Figure S7. Analysis of BET inhibitor-induced cytotoxicity in a panel of solid tumor cell lines. **(a)** Correlation of I-BET726 sensitivity as measured by Y_{min}-T_0 to relative RNA expression of *MYC* (left) or *MYCN* (right) in the neuroblastoma cell line panel. p-values from two-tailed Pearson correlation analyses are indicated for each comparison. **(b)** Correlation of I-BET151 sensitivity as measured by Y_{min}-T_0 to relative RNA expression of *MYC* (left) or *MYCN* (right) as described in (a).

Figure S7

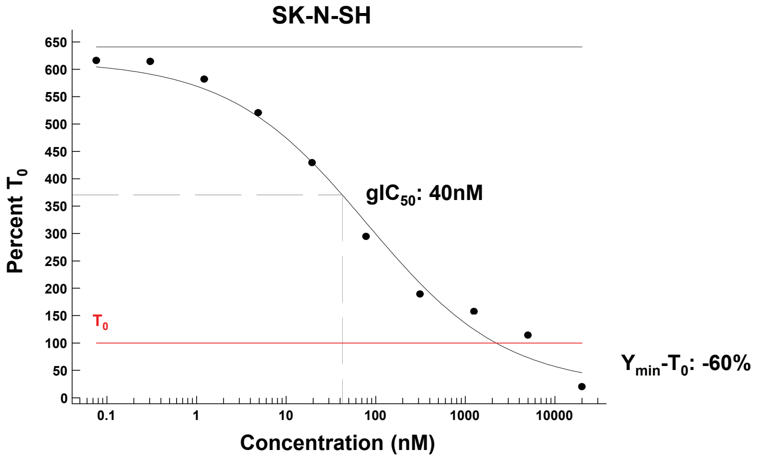

Figure S8. Concentration response curve for I-BET726 from a 6 day growth-death assay in SK-N-SH. Solid black horizontal line indicates growth in DMSO-treated controls. Solid red line indicates T_0 (100%). gIC_{50} and Y_{min}-T_0 values are indicated. Data presented as the average of two independent curves from a single experiment, and is representative of data from four independent biological replicates.

Figure S8

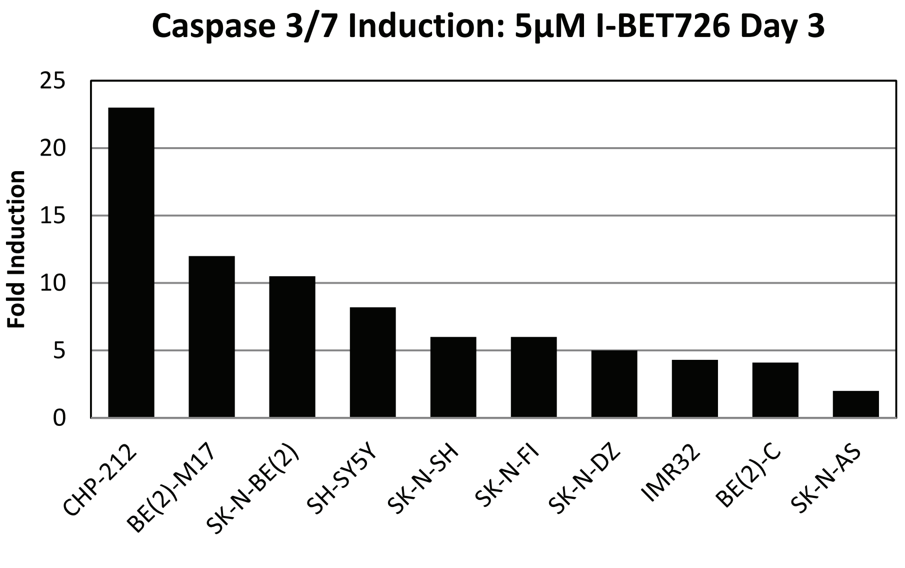

Figure S9. Analysis of effects of I-BET726 on Caspase 3/7 induction in neuroblastoma cell lines. Fold induction of Caspase 3/7 activity relative to DMSO in the indicated neuroblastoma cell lines following treatment with 5 µM I-BET726 for three days. Data represents the average of two independent replicates.

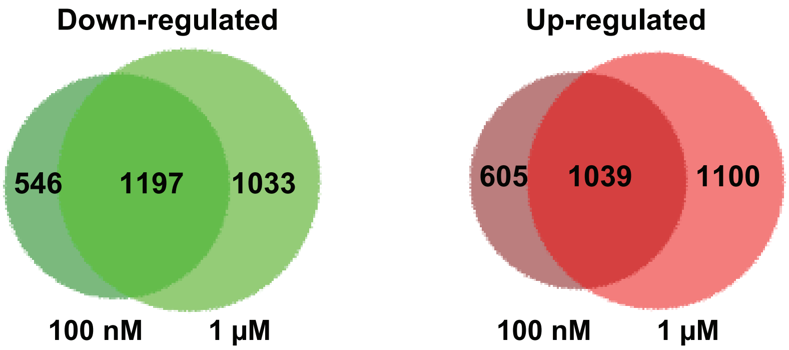

Figure S10. Venn diagrams indicating the degree of overlap between 100 nM and 1 µM I-BET726 treatment in the SK-N-SH cell line from the gene expression profiling experiment.

Figure S10

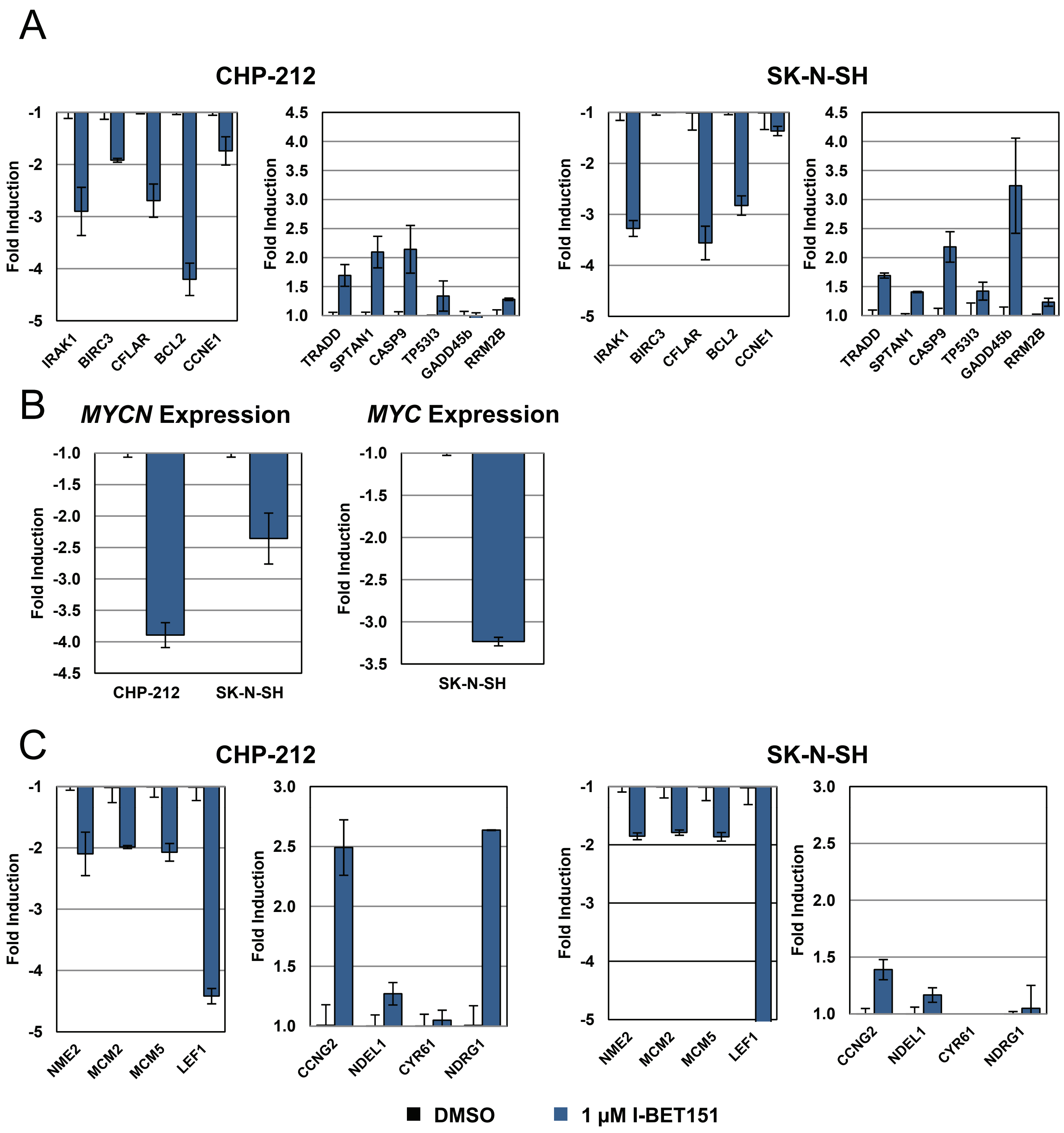

Figure S11. qPCR analyses following treatment of CHP-212 or SK-N-SH with DMSO or 1 µM I-BET151 for 16 hours. **(a)** qPCR analysis of genes validated in Figure 3E. Data represent mean value ± standard deviation for two independent biological replicates. **(b)** qPCR analysis of *MYCN* and *MYC* expression as described in (a). **(c)** qPCR analysis of N-Myc network genes validated in Figure 4D as described in (a).

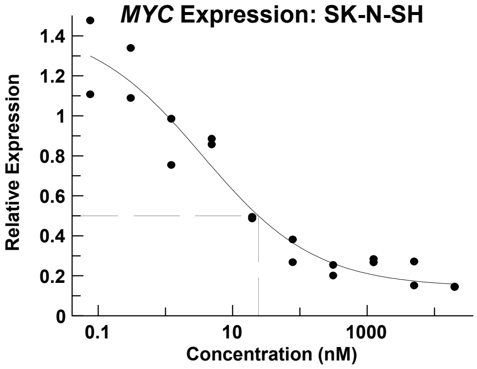

Cell Line	*MYC* IC_{50}	Percent Maximal Inhibition
SK-N-SH	35 nM	85%
SK-N-AS	1000 nM	55%
SK-N-FI	>20000 nM	15%

Figure S12. Left: Analysis of *MYC* gene expression in the SK-N-SH cell line following treatment with I-BET726. qPCR determination of *MYC* RNA expression following 24 hour treatment with a concentration titration of I-BET726. Data were normalized to expression of *GAPDH*, and are presented as expression relative to DMSO-treated control samples. Data shown were from a single experiment representative of typical results. Right: Table of average IC_{50} values and percent inhibition for *MYC* expression following 24 hour treatment with I-BET726.

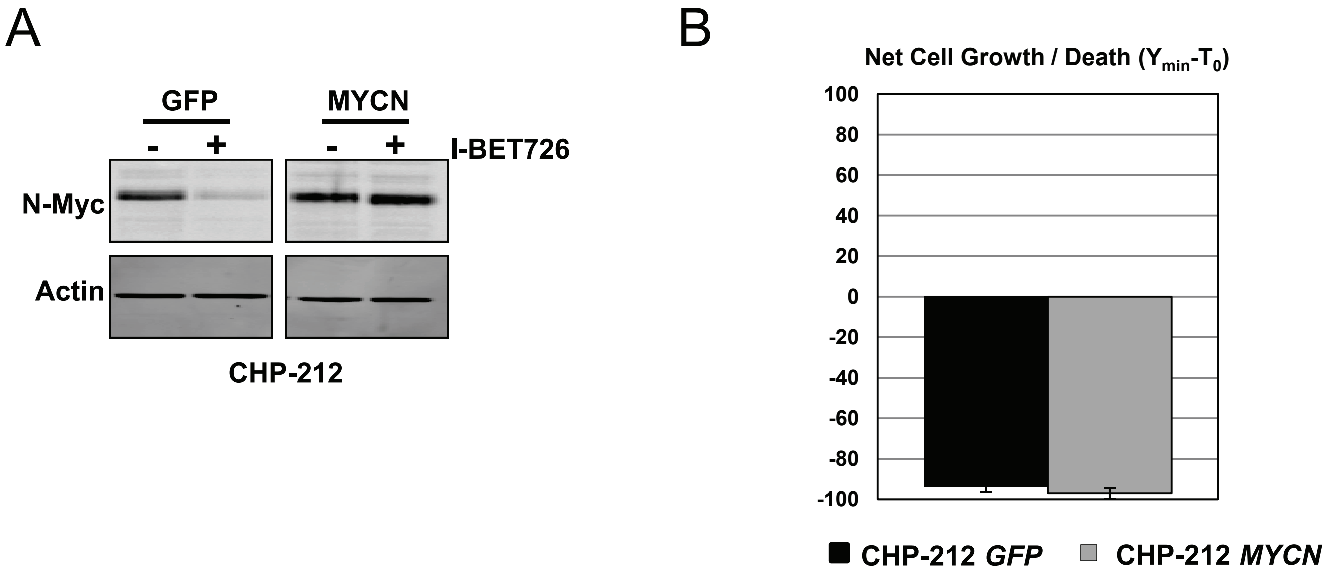

Figure S13. (**a**) Western blot analysis of N-Myc expression in CHP-212 cells overexpressing GFP (left) or N-Myc (right) from a lentiviral expression vector following treatment with DMSO or 1 μM I-BET726 for 48 hours. (**b**) Y_{min}-T_0 values determined for *GFP*- and *MYCN*-transduced CHP-212 cells following 6 days treatment with I-BET726. Data represent mean value ± standard deviation for three independent biological replicates.

Figure S13

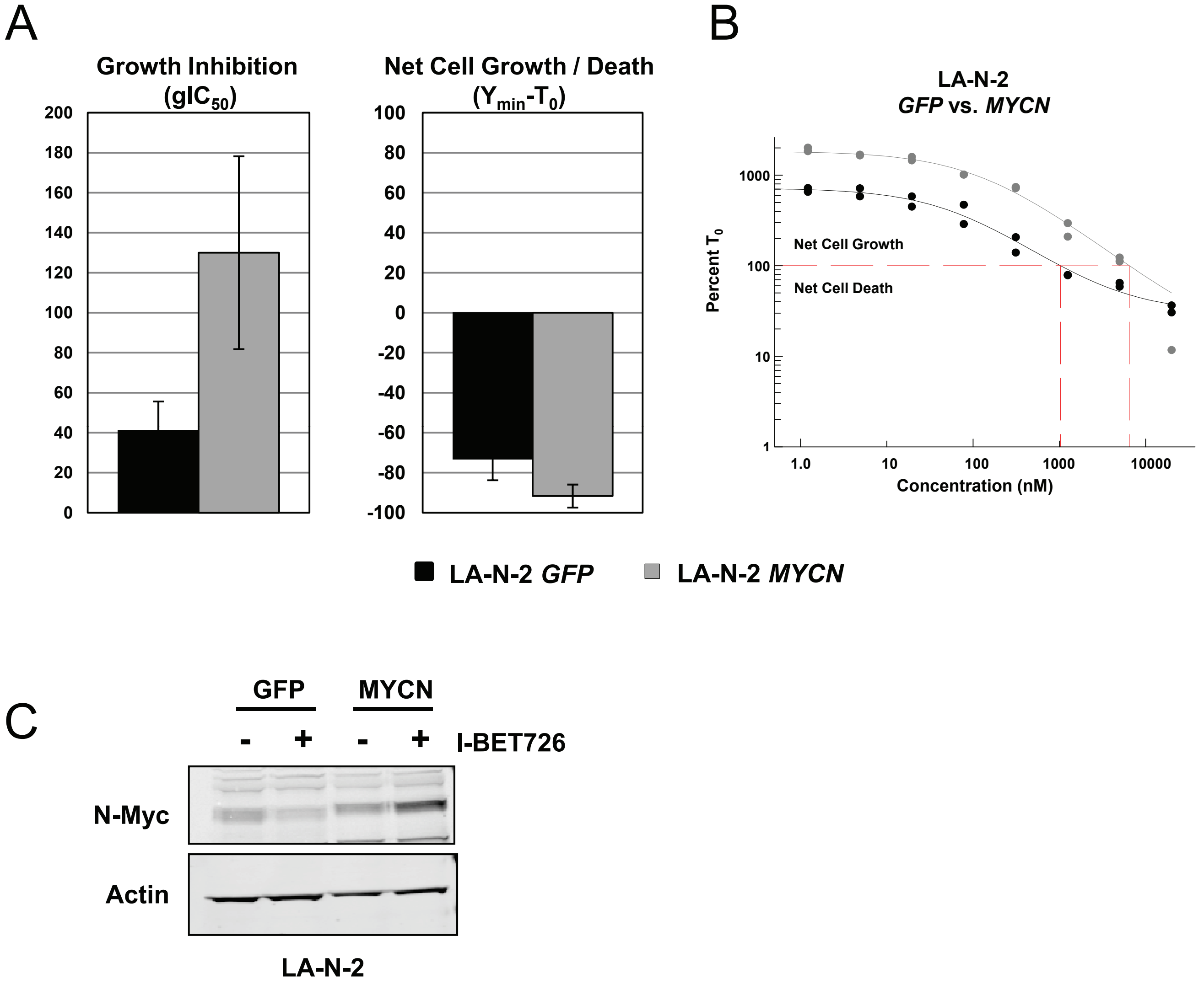

Figure S14. (**a**) gIC_{50} (left) and Y_{min}-T_0 (right) values following I-BET726 treatment in the LA-N-2 cell line overexpressing *GFP* or *MYCN*. Data represent mean value ± standard deviation for three independent biological replicates. p-values determined from t-tests for gIC_{50} and Y_{min}-T_0 are 0.064 and 0.053, respectively. (**b**) Concentration response curves for *GFP* or *MYCN*–overexpressing LA-N-2 cells from 6 day growth-death assay. Horizontal line indicates T_0 measurement (set to 100%). Data shown were from a single experiment representative of typical results. (**c**) Western blot of N-Myc and Actin expression following I-BET726 treatment for 48 hours in the LA-N-2 cell line overexpressing the *GFP* or *MYCN* gene.

Figure S14

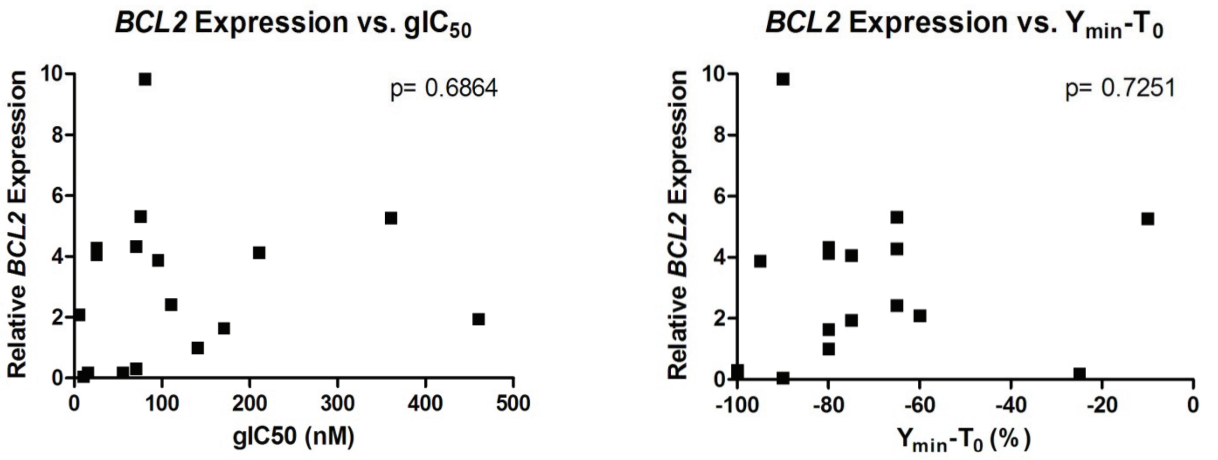

Figure S15. Correlation of I-BET726 sensitivity as measured by gIC_{50} (left) and Y_{min}-T_0 (right) to relative RNA expression of *BCL2* in the neuroblastoma cell line panel as described in Figure S3C. p-values are indicated for each comparison.

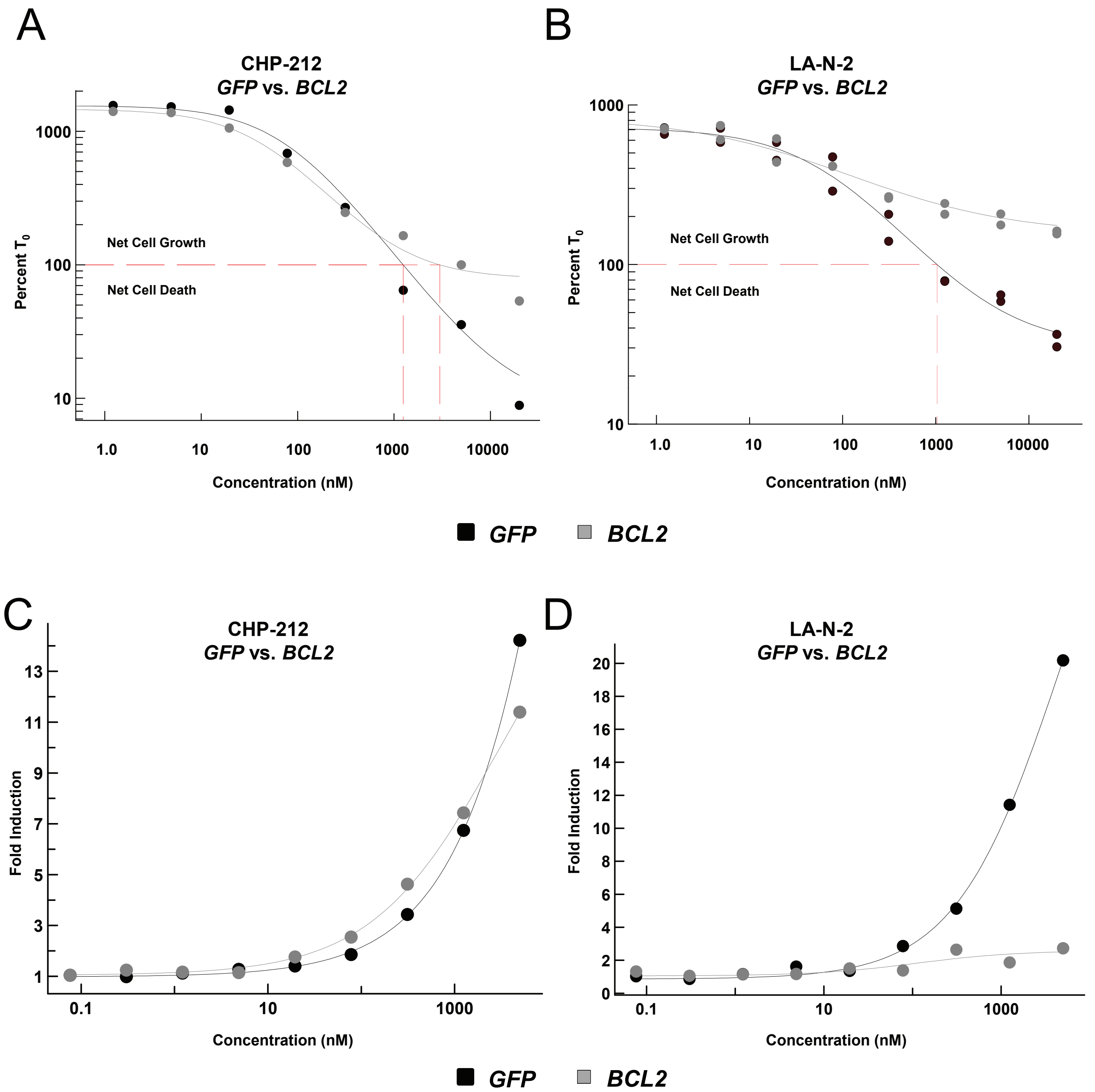

Figure S16. (**a**) Concentration response curves for I-BET726 in *GFP* or *BCL2*–overexpressing CHP-212 cells from a 6 day growth-death assay. Horizontal line indicates T_0 measurement (set to 100%). Data shown were from a single experiment representative of typical results. (**b**) Concentration response curves for *GFP* or *BCL2*–overexpressing LA-N-2 cells as described in (a). (**c**) Caspase induction in *GFP* or *BCL2*-overexpressing CHP-212 cells following treatment with a titration of I-BET726 for six days. Data is presented as fold induction over DMSO controls, following normalization to total cell number as measured by CellTiter-Glo. Data shown were from a single experiment representative of typical results. (**d**) Caspase induction in *GFP* or *BCL2*-overexpressing LA-N-2 cells as described in (c).

Figure S16

Blood Concentrations of I-BET726 (ng/ml)		
**Dose**	**SK-N-AS**	**CHP-212**
5 mg/kg Day 1	170 +/- 70	265 +/- 95
5 mg/kg Day 8	65 +/- 35	265 +/- 205
15 mg/kg Day 1	785 +/- 425	1390 +/- 630
15 mg/kg Day 8	895 +/- 605	980 +/- 380
**Tumor Concentrations of I-BET726 (ng/ml)**		
**Dose**	**SK-N-AS**	**CHP-212**
5 mg/kg Day 1	120 +/- 25	140 +/- 50
5 mg/kg Day 8	90 +/- 20	130 +/- 95
15 mg/kg Day 1	705 +/- 205	485 +/- 140
15 mg/kg Day 8	520 +/- 195	700 +/- 285

Figure S17. Blood and tumor concentrations of I-BET726 in the SK-N-AS and CHP-212 xenograft models, measured on Day 1 and Day 8 of the study. Data represents the mean ± SD for each dosing group (n=3).
