# Supplementary material for: BET Inhibition Silences Expression of MYCN and BCL2 and Induces Cytotoxicity in Neuroblastoma Tumor Models
Source: PLoS One. 2013 Aug 23;8(8):e72967. doi: 10.1371/journal.pone.0072967 (PMC3751846; doi:10.1371/journal.pone.0072967)
Supplement: Methods S1 — (DOCX) [file pone.0072967.s007.docx]

**Supplemental Methods**

**Antibodies**Antibodies used in Western blotting and ChIP experiments are as follows: Actin (A-2066, Sigma-Aldrich, St. Louis, MO); Bcl-2 (ab7973, Abcam, Cambridge, MA); BRD4 (ab84776, Abcam); c-Myc (#9402, Cell Signaling Technology, Danvers, MA); N-Myc (ab16898, Abcam); rabbit IgG (49-2024, Life Technologies, Grand Island, NY); Tubulin (T-9026, Sigma-Aldrich). Secondary antibodies labeled with IRDye®680 and IRDye®800 were from LICOR Biosciences (Lincoln, NE).

**Crystal Structure of BRD4-BD1/I-BET726 Complex**

E. coli expressed His-tagged BRD4-BD1(44-168) was generated as previously described [1,2]. The protein was at 9.7mg/ml in 10mM HEPES pH7.5, 100mM NaCl and purified to homogeneity using a HisTrap column followed by gel filtration, Tev protease cleavage and gel filtration using a Sephadex 75 column. I-BET726 was added to the protein at 3:1 excess and spun prior to co-crystallization in 120nl +120nl sitting drops at 20°C using MRC plates over a solution of 20% PEG3350, 0.2M NaK,tartrate, 0.1M bistrispropane pH 6.5. Crystals were briefly transferred into cryo buffer of the well solution with 20% glycerol added before flash freezing in liquid nitrogen. Data from a single crystal was collected on an in house RIGAKU FRE+ SUPERBRIGHT/A200 system and processed to 1.509 Å using XDS (Acta Cryst. (2010) D66, 133-144) and SCALA(CCP4). Molecular replacement solution was performed with 2oss.pdb using Phaser (CCP4). The P2_1_2_1_2_1_ cell (a=b=g=90°, a=41.894Å b=59.542Å, c=109.195Å) has a single molecule in the ASU. Model building was performed using Coot and refined using refmac (CCP4, Acta Cryst. (1994) D50: 760-763). The difference density was excellent and I-BET726 ligand could be unambiguously modelled. The statistics for the data collection and refined coordinates are listed below. The final model (R/R_free_ : 15.6%/18.5%) is deposited in the Protein Data Bank (http://www.rcsb.org/pdb/home/home.do) under the accession code 4bjx.pdb.

| Data collection and refinement statistics (**Molecular replacement**) | |
| --- | --- |
|  |  |
| (collection on a single crystal) | BRD4-BD1/ I-BET726 |
| **Data collection** |  |
| Space group | P2_1_2_1_2_1_ |
| Cell dimensions |  |
| *a*, *b*, *c* (Å) | 44.780 47.906 58.757 |
| *a*, *b*, *g* (°) | 90.0, 90.0, 90.0 |
| Resolution (Å) | 58.76-1.59 (1.68-1.59) * |
| *R*_merge_ | 0.022 (0.053) |
| *I*/σ*I* | 55.3 (21.1) |
| Completeness (%) | 98.6 (91.2) |
| Redundancy | 6.0 (3.0) |
|  |  |
| **Refinement** |  |
| Resolution (Å) | 58.76-1.59 |
| No. reflections | 104056 (6830) |
| No. uniq reflections | 17351 (2285) |
| *R*_work/_ *R*_free_ | 0.156/0.185 |
| No. atoms | 1415 |
| Protein | 1087 |
| Ligand/ion | 31/0 |
| Water | 297 |
| B-factors |  |
| Protein | 9.86 |
| Ligand/ion | 9.05/0 |
| Water | 22.8 |
| R.m.s deviations |  |
| Bond lengths (Å) | 0.005 |
| Bond angles (º) | 1.06 |
| *Highest resolution shell is shown in parenthesis. | |

**Determination of BET Protein Binding Affinities to I-BET726**

For determination of binding affinities to BET protein bromodomains, I-BET726 was titrated against truncates containing both BD1 and BD2 of BRD2 (10nM), BRD3 (10nM), and BRD4 (10nM) in 50 mM HEPES pH7.5, 150 mM NaCl, 5% Glycerol, 1 mM DTT and 1 mM CHAPS in the presence of an Alexa 647 derivative (50 nM) of fluorescent ligand [1]. After equilibrating for 1 h, the bromodomain protein: ligand interaction was detected using Time Resolved Fluorescence Resonance Energy Transfer (TR-FRET) following the addition of 1.5 nM europium chelate labeled anti-6His antibody (Perkin Elmer, Waltham, MA). Plates were read using an Envision Plate reader (λ_EX_ = 337 nm, λ_EM_ = 615 nm, λ_EM_ = 665 nm; dual dichroic = 400 nm & 630 nm). These data were fitted to a four parameter IC_50_ model using Graphit data analysis software.

**Selectivity Profiling**

Thermal shift assays of bromodomain‐containing proteins in the presence of I-BET726 or DMSO control were performed as described previously [3]. The following bromodomain constructs were tested: ATAD2, BAZ2B, PCAF, SMARCA2, SP140, TAF1, CREBBP, BRD4-BD1, BRD4-BD2, BRD4-BD1-BD2, BRD2-BD1-BD2.

**Isothermal Titration Calorimetry**

For BRD4, ITC titrations were carried out using a Microcal VPITC in 50mM Hepes, pH 7.5, 150mM NaCl at 25^o^C. 9µM of His-tagged-BRD4 (1-477) tandem bromodomain was placed in the cell and 200µM ligand (I-BET726) was titrated into this to achieve a final excess ~ 4:1 ligand:protein. The data was fitted within Origin (Microcal version) to give the following parameters: stoichiometry of 1.93, a K_D_ of 4.4±0.9nM, ΔH -15.9±0.08kcal/mol, ΔS -15.2 cal/mol/deg.

For CREBBP, ITC titrations were carried out using a GE autoITC200 in 50mM Hepes, pH 7.4, 150mM NaCl at 25^o^C. 44µM of the single bromodomain of CREBBP was placed in the cell and 500µM ligand (I-BET726) was titrated into this to achieve a final excess ~ 2.2:1 ligand:protein. The data was fitted within Origin (Microcal version) to give the following parameters: stoichiometry of 1.1, a K_D_ of 6.3±0.5uM, ΔH -4.35±0.08kcal/mol, ΔS 9.2 cal/mol/deg.

**RNA Expression Analysis**

For *MYC* and *MYCN* expression analyses, exponentially growing cells were harvested and lysed in Trizol reagent (Life Technologies). Chloroform and RNase-free water was added to each sample, and phase separation was performed by applying samples to MaXtract High Density tubes (Qiagen, Valencia, CA), following the manufacturer’s recommendations. RNA was purified from the aqueous phase using the RNeasy mini kit (Qiagen) following the manufacturer’s instructions. cDNA was generated using the High Capacity cDNA Reverse Transcription kit (Life Technologies) following the manufacturer’s instructions. Gene expression was measured via TaqMan analysis on an Applied Biosystems ViiA7 real-time PCR machine. Relative expression was calculated compared to cDNA obtained from normal tissue controls, using *HPRT* as an internal control. *BRD2*, *BRD3*, *BRD4*, and *BCL2* expression analyses were performed as described above, and relative expression was calculated compared to expression in the BE(2)-C cell line.

Confirmation of gene expression changes from the microarray experiment and mouse pharmacodynamic studies were performed as described above. Relative expression was calculated compared to cDNA obtained from vehicle-treated controls, using *GAPDH* as an internal control.

All TaqMan assays for qPCR analysis were obtained from Life Technologies. Specific assay ID numbers or catalog numbers are listed below.

| **Gene** | **TaqMan Assay** |  | **Gene** | **TaqMan Assay** |
| --- | --- | --- | --- | --- |
| BCL2 | Hs00608023_m1 |  | IRAK1 | Hs01018347_m1 |
| BIRC3 | Hs00985031_g1 |  | LEF1 | Hs01547250_m1 |
| BRD2 | Hs01121984_m1 |  | MCM2 | Hs01091564_m1 |
| BRD3 | Hs00201284_m1 |  | MCM5 | Hs00198823_m1 |
| BRD4 | Hs00293232_m1 |  | MYC | Hs99999003_m1 |
| CASP9 | Hs00154261_m1 |  | MYCN | Hs00232074_m1 |
| CCNE1 | Hs01026536_m1 |  | NDEL1 | Hs00229366_m1 |
| CCNG2 | Hs00171119_m1 |  | NDRG1 | Hs00608387_m1 |
| CD82 | Hs01017982_m1 |  | NME2 | Hs00897133_g1 |
| CFLAR | Hs00153439_m1 |  | RCHY1 | Hs00295839_m1 |
| CYR61 | Hs00998500_g1 |  | RPL19 | Hs02338565_gH |
| EXOG | Hs00270782_m1 |  | RRM2B | Hs00968432_m1 |
| GADD45b | Hs00169587_m1 |  | SPTAN1 | Hs00162203_m1 |
| GAPDH | 4352934E |  | TP53IP | Hs00936520_m1 |
| GUS | 4326320E |  | TRADD | Hs00182558_m1 |
| HPRT | Hs02800695_m1 |  |  |  |
|  |  |  |  |  |

**Chromatin Immunoprecipitation (ChIP)**

Cells were cross-linked with 1% formaldehyde for 10 minutes at room temperature and then quenched by incubation with 0.125 M glycine for an additional 10 minutes. Cells were then harvested and washed, and nuclear extraction was performed by sequential resuspension of cell pellets in CEBN and CEB buffer (CEB: 10 mM HEPES, pH 7.8, 10 mM KCl, 1.5 mM MgCl_2_, 0.34 M Sucrose, 10% Glycerol; CEBN: CEB + 0.2% NP-40). Nuclear pellets were then lysed in SDS lysis buffer (Millipore, Billerica, MA), and DNA was fragmented to a mean fragment size of 500 bp using a Bioruptor (Diagenode, Denville, NJ). Equivalent amounts of chromatin from each sample were then immunoprecipitated with ChIP antibodies in ChIP dilution buffer (Millipore) overnight at 4°C. Antibody-protein complexes were then collected using a mixture of Protein A and Protein G Dynabeads (Life Technologies) pre-blocked with 0.5% BSA. Beads were washed with low salt immune complex wash buffer, high salt immune complex wash buffer, LiCl immune complex wash buffer (Millipore), and 1x TE (Promega), followed by elution in elution buffer (50 mM Tris-HCL, pH 8.0, 10 mM EDTA, 1% SDS). Eluted DNA was reverse cross-linked, treated with RNase A and proteinase K, and purified using the QIAquick PCR purification kit (Qiagen) following the manufacturer’s protocol. Immunoprecipitated DNA and input controls were then analyzed on an Applied Biosystems ViiA 7 real-time PCR machine, using the primer sets listed below.

| **Gene** | **Assay** | **Source** |
| --- | --- | --- |
| BCL2 promoter | GPH1020168(-)01A | Qiagen |
| MYCN promoter | GPH1007307(-)01A | Qiagen |
| Chr. 12 Intergenic | GPH100001C(-)01A | Qiagen |

**Lentiviral Vector Construction of pLEX-BCL2 and pLEX-MYCN**

The coding sequence of *BCL2* was amplified from pCMV6-XL4-BCL2 (Origene, Rockville, MD) using Phusion Hot Start II High Fidelity DNA Polymerase (Thermo Fisher Scientific, Waltham, MA USA) with forward primer 5’-CACCGACTCTACTAGAGGATCCGCCACCATGGCGCACGCTGGGAGAACAGGGTACGATAACCGGGAG-3’ and reverse primer 5’ GACGCGTCGGGCCCTCTAGACTCGAGTCACTTGTGGCCCAGATAGGCACCCAGGGTG-3’.

# The coding sequence of *MYCN* was amplified from pCMV6-XL4 –MYCN (Origene) using Phusion Hot Start II High Fidelity DNA Polymerase (Thermo Fisher Scientific) with forward primer 5’- GACACCGACTCTACTAGAGGATCCGCCACCATGCCGAGCTGCTCCACGTCCACCATGC-3’ and reverse primer 5’ CGGGCCCTCTAGACTCGAGCTAGCAAGTCCGAGCGTGTTCAATTTTCTTTAGC-3’.

Amplified DNA was purified by QIAquick Gel Extraction Kit (Qiagen)  and inserted into 5’ BamHI and 3’ XhoI sites of LentiORF pLEX-MCS using In-Fusion® HD Cloning System (Clontech, Mountain View, CA). Incorporation of BCL2 and MYCN were verified by sequencing and referred to as pLEX-BCL2 and pLEX-MYCN, respectively.

**Lentivirus Production**

HEK293T cells were grown in a humidified incubator at 5% CO_2_ and 37°C, and maintained in DMEM-high glucose supplemented with 10% fetal bovine serum and propagated on CellBind™ tissue culture ware (Corning, Tewksbury, MA). Lentivirus production was performed by co-transfecting HEK293T cells in reduced serum (DMEM-high glucose supplemented with 5% FBS) with ExpressIN™ transfection reagent (Thermo Fisher Scientific) complexed with viral components VSVG, gag-pro, and Tat/Rev (Thermo Fisher Scientific) and pLEX-BCL2 or pLEX-MYCN. Viral supernatants were harvested 72 hrs post-transfection by centrifugation at 2,500 rpm for 15 minutes at room temperature. *BCL2* and *MYCN* clarified viral supernatant were titered using an ELISA measuring viral associated p24 concentrations relative to a p24 standard curve (Cell Biolabs, San Diego, CA).

**Lentiviral Transduction Experiments**

Infections were carried out in serum-free RPMI-1640 media at an M.O.I. of 3 over a period of 24 hours. Media containing virus was then removed and replaced with normal growth media. The following day, transduced cells were selected by the addition of 1 µg/ml puromycin to the growth media. Following 96 hours of selection in puromycin, cells were trypsinized, counted and plated for growth-death analysis, cell cycle analysis, or Western blot in normal growth media containing 0.125 µg/ml puromycin. Assays were carried out as described above.

**References**

1. Chung CW, Coste H, White JH, Mirguet O, Wilde J, et al. (2011) Discovery and characterization of small molecule inhibitors of the BET family bromodomains. J Med Chem 54: 3827-3838.

2. Nicodeme E, Jeffrey KL, Schaefer U, Beinke S, Dewell S, et al. (2010) Suppression of inflammation by a synthetic histone mimic. Nature 468: 1119-1123.

3. Dawson MA, Prinjha RK, Dittmann A, Giotopoulos G, Bantscheff M, et al. (2011) Inhibition of BET recruitment to chromatin as an effective treatment for MLL-fusion leukaemia. Nature 478: 529-533.
